# Cathepsin L-like Cysteine Proteinase Genes Are Associated with the Development and Pathogenicity of Pine Wood Nematode, *Bursaphelenchus xylophilus*

**DOI:** 10.3390/ijms20010215

**Published:** 2019-01-08

**Authors:** Qi Xue, Xiao-Qin Wu, Wan-Jun Zhang, Li-Na Deng, Miao-Miao Wu

**Affiliations:** 1 Co-Innovation Center for Sustainable Forestry in Southern China, College of Forestry, Nanjing Forestry University, Nanjing 210037, China; xq2159@163.com (Q.X.); 18251980171@163.com (W.-J.Z.); 13675164953@163.com (M.-M.W.); 2 Jiangsu Key Laboratory for Prevention and Management of Invasive Species, Nanjing Forestry University, Nanjing 210037, China; 3 Yancheng Institute of Technology, School of Ocean and Biological Engineering, Yancheng 224051, China; denglina121@163.com

**Keywords:** *Bursaphelenchus xylophilus*, cathepsin L, gene expression, development, pathogenicity

## Abstract

The pine wood nematode (PWN), *Bursaphelenchus xylophilus*, is the pathogen of pine wilt disease (PWD), resulting in huge losses in pine forests. However, its pathogenic mechanism remains unclear. The cathepsin L-like cysteine proteinase (CPL) genes are multifunctional genes related to the parasitic abilities of plant-parasitic nematodes, but their functions in PWN remain unclear. We cloned three *cpl* genes of PWN (*Bx-cpls*) by rapid amplification of cDNA ends (RACE) and analyzed their characteristics using bioinformatic methods. The tissue specificity of *cpl* gene of PWN (*Bx-cpl*) was studied using in situ mRNA hybridization (ISH). The functions of *Bx-cpl*s in development and pathogenicity were investigated using real-time quantitative PCR (qPCR) and RNA interference (RNAi). The results showed that the full-length cDNAs of *Bx-cpl-1*, *Bx-cpl-2*, and *Bx-cpl-3* were 1163 bp, 1305 bp, and 1302 bp, respectively. *Bx-cpl*s could accumulate specifically in the egg, intestine, and genital system of PWN. During different developmental stages of PWN, the expression of *Bx-cpl*s in the egg stage was highest. After infection, the expression levels of *Bx-cpl*s increased and reached their highest at the initial stage of PWD, then declined gradually. The silencing of *Bx-cpl* could reduce the feeding, reproduction, and pathogenicity of PWN. These results revealed that *Bx-cpl*s play multiple roles in the development and pathogenic processes of PWN.

## 1. Introduction

The pine wood nematode (PWN), *Bursaphelenchus xylophilus*, is the causal agent of pine wilt disease (PWD). It has been detected in North America (USA, Canada, and Mexico) [1,2], East Asia (Japan, China, and Korea) [3,4,5], Europe (Portugal and Spain) [6,7], and Nigeria [8]. The disease has been unquestionably a major threat to forest ecosystems worldwide and has caused great losses in China. However, the pathogenic mechanism of *B. xylophilus* remains unclear.

With the development of biotechnology, the expressed sequence tags (ESTs), genome, transcriptome, and secretome of *B. xylophilus* have been analyzed, highlighting several groups of genes putatively related to its pathogenicity [9,10,11,12,13]. Cellulase genes [14,15], pectatelyase genes [16,17], expansin-like genes [18,19], the venom allergen-like protein gene [20], and cytochrome P450 genes [21] have been studied and identified as pathogenesis-related genes. The functions of other putative pathogenesis-related genes of *B. xylophilus* still need to be identified.

It is believed that peptidases are essential for parasite development and in the most critical situations of parasite–host interactions. Peptidases comprise a large class of hydrolytic enzymes in parasites [22]. Of these, the cysteine peptidases are the class that covers virtually all functions that involve peptidases in parasitic helminths (including trematodes, cestodes, and nematode parasites) [23]. Cathepsin L is a type of cysteine peptidase belonging to the papain family and has been comprehensively studied in many parasitic helminths [24]. In free-living and parasite nematodes of humans and animals, the cathepsin L proteinases are involved in pivotal functions, such as tissue penetration, nutrition, immune evasion, and eggshell formation, though little is known of their precise functions [25]. As with animal parasite counterparts, nematodes that infect plants may require proteinases for egg hatching, larval molting, tissue penetration, and feeding. Urwin et al. [26] were the first team to clone a cathepsin L-like proteinase (CPL) from *Heterodera glycines*. To date, a number of *cpl* genes from plant parasitic nematodes including *Bursaphelenchus*, *Globodera*, *Heterodera*, *Meloidogyne*, and *Rotylenchulus* have been cloned, but their functions are seldom reported formally. The *cpl* gene of *Meloidogyne incognita* (*Mi-cpl-1*) encodes a digestive enzyme which is consistent with feeding [27,28]. In addition, *Mi-cpl-1* can affect *M. incognita* development and play a crucial role in plant–nematode interactions [28,29,30]. The *cpl* gene in *M. hispanica* was also identified and characterized as a parasitism gene [31]. In *B. xylophilus*, two *cpl* genes (ACH69776.1, ACH56225.1) have been cloned, but their functions have not yet been investigated. In this study, the full-length cDNA of three novel *cpl* genes—*Bx-cpl-1*, *Bx-cpl-2*, and *Bx-cpl-3*—were cloned using 3′ and 5′ rapid amplification of cDNA ends (RACE). The expressions of *Bx-cpls* in *B. xylophilus* at different developmental and pathogenic stages associated with PWD were analyzed by qPCR. The roles of *Bx-cpls* in reproduction and pathogenicity were verified through RNA interference (RNAi). These results provide useful information to better understand the functions of *cpl*s in *B. xylophilus* and to elucidate the molecular pathogenic mechanism.

## 2. Results

### 2.1. Cloning and Sequence Analysis of Three Cathepsin L-Like Cysteine Proteinase Genes from B. xylophilus

**Figure 1:**
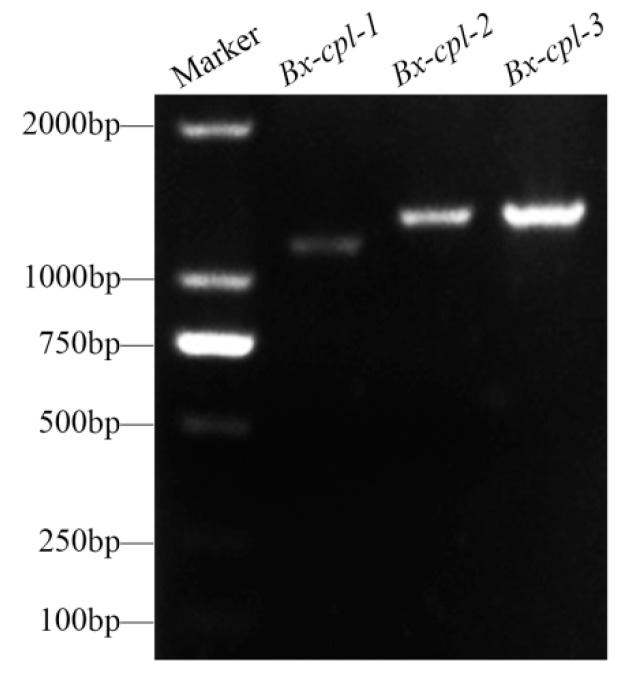
Bands of *Bx-cpls* full-length cDNA sequences after gel electrophoresis.

The complete nucleotide sequence of *Bx-cpl-1* had 1163 bp (Figure 1), including an 18 bp 5′ untranslated region (UTR), a 1074 bp open reading frame (ORF), and a 71 bp 3′ UTR. It encodes a protein of 357 amino acid residues (Figure S1A). The full-length cDNA of *Bx-cpl-2* had 1305 bp (Figure 1), comprising a 48 bp 5′ UTR, an 1185 bp ORF encoding 394 amino acid residues, and a 72 bp 3′ UTR (Figure S1B). The full-length cDNA of *Bx-cpl-3* was 1302 bp (Figure 1), including a 51 bp 5′ UTR, a 63 bp 3′ UTR, and an 1188 bp ORF encoding 395 amino acids (Figure S1C). Compared to the genome data available on WormBase Parasite (BioProject PRJEA64437), the genomic locations of *Bx-cpl-1*, *Bx-cpl-2*, and *Bx-cpl-3* were at scaffold01141 191,468 to 192,800 with three introns, scaffold00813 265,318 to 266,698 with two introns, and scaffold01147 920,147 to 921,523 with two introns, respectively (Figure S2).

**Figure 2:**
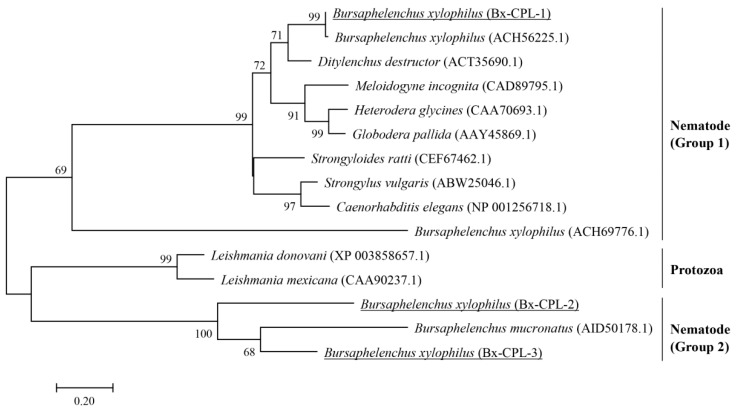
Phylogenetic relationships of cathepsin L-like cysteine proteinases (CPLs). The phylogram was constructed based on amino acid sequences to determine the evolutionary relationships among 15 CPL proteins from different species using MEGA 7. The numbers below the branches indicate the bootstrap values, which were calculated from 1000 replicates. The GenBank accession numbers of the sequences are in brackets. *B. xylophilus* CPLs (Bx-CPL-1, Bx-CPL-2 and Bx-CPL-3) are underlined. Distance scale = 0.2.

The results of Blastp showed that some CPL proteins have a relatively high level of identity with the CPLs of *B. xylophilus*. On this basis, amino acid sequences from Nematode and Protozoa which showed relatively high homology with the predicted amino acid sequences from *B. xylophilus* were selected and downloaded from NCBI. The phylogenetic tree was constructed by the maximum likelihood method with a WAG model with gamma-distributed rates based on the amino acid sequences of CPL proteins (Figure 2). Three CPL proteins of *B. xylophilus* were divided into two nematode groups. The *Bx-cpl-1* deduced protein, Bx-CPL-1, is closely related to plant parasitic nematodes *Ditylenchus destructor*, *Meloidogyne incognita*, *Heterodera glycines*, *Globodera pallida*, and *B. xylophilus* in particular (ACH56225.1). However, the *Bx-cpl-2* and *Bx-cpl-3* deduced proteins, Bx-CPL-2 and Bx-CPL-3, were highly linked to *B. mucronatus* (AID50178.1) and even the CPLs in Protozoa, rather than the other CPLs in Nematode (Figure 2).

### 2.2. Localization of Bx-cpl in B. xylophilus

**Figure 3:**
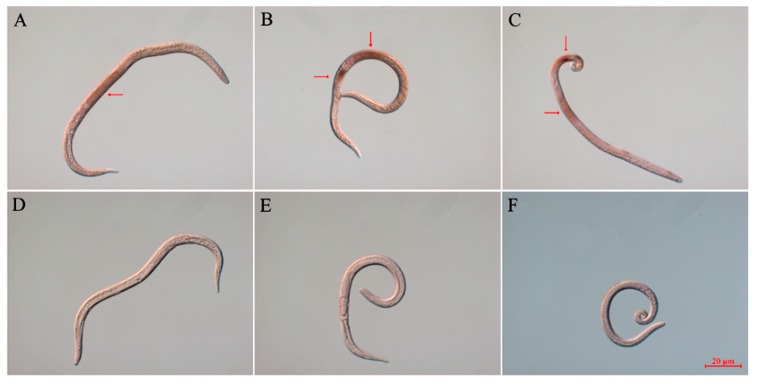
Localizations of *Bx-cpl*s mRNA by in situ hybridization (ISH). *Bx-cpl* was expressed in PWNs in the intestine of females (**A**); the intestine and egg of females (**B**); and the intestine and seminal vesicle of males (**C**). The control groups showed no signals (**D**–**F**). The red arrows point to the hybridization signals. The scale bars are 20 µm.

In situ hybridization (ISH) was used to analyze the tissue specificity of *Bx-cpl* transcription. The localizations of the three *Bx-cpls* were similar. The digoxigenin (DIG)-labeled antisense RNA probe of *Bx-cpl* generated clear signals in the intestine and egg of females (Figure 3A,B), and intestine and seminal vesicle of males of *B. xylophilus* (Figure 3C). No signals were observed in the control group with the sense probes.

### 2.3. Expression of Bx-cpl at PWN Developmental Stages

**Figure 4:**
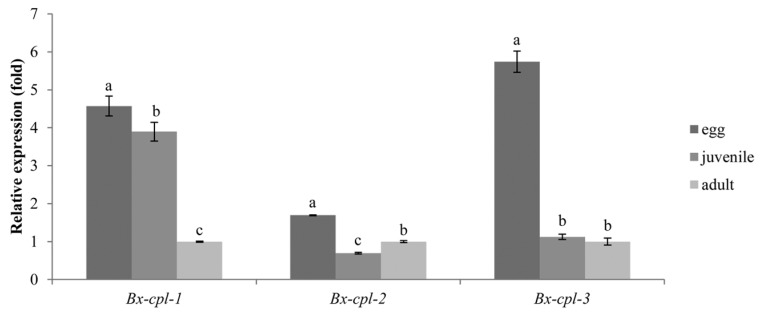
Relative expression levels of *Bx-cpl*s at different developmental stages of *B. xylophilus*. The bars indicate standard errors, and different letters indicate significant differences (*p* < 0.05) among the different nematode stages (egg, juvenile, and adult).

All *Bx-cpl*s showed relatively high transcript levels in the egg stage. The *Bx-cpl-1* expression was significantly lower in adults than in juveniles (*p* < 0.05), and *Bx-cpl-2* expression was the opposite. There was no significant difference in *Bx-cpl-3* expression between juveniles and adults (*p* > 0.05) (Figure 4).

### 2.4. Expression of Bx-cpl at PWD Development Stages

**Figure 5:**
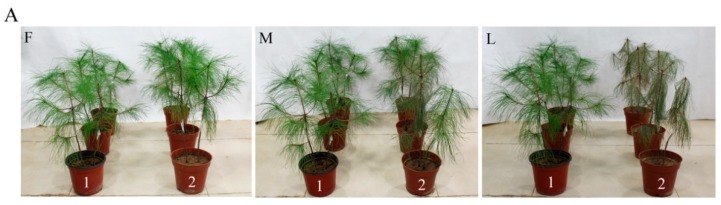

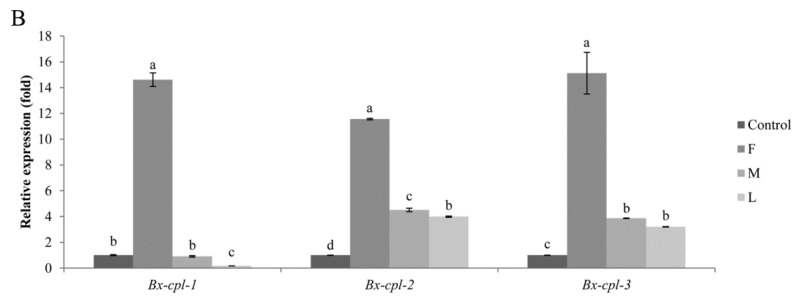
Symptoms in *P. massoniana* after inoculation with nematodes: (**A**) First stage of pine wilt disease (PWD) (F), middle stage of PWD (M), and last stage of PWD (L). Pines inoculated with ddH_2_O (1); Pines inoculated with *B. xylophilus* (2). Relative expression levels of *Bx-cpl*s at PWD development stages (**B**). The bars indicate standard errors, and different letters indicate significant differences (*p* < 0.05).

After infection of pine seedlings with *B. xylophilus*, all three *Bx-cpl*s were found to be upregulated and reached the highest expression level at the first stage of PWD. Then, their expression declined and reached its lowest level at the late stage (Figure 5). These results indicate that the three *Bx-cpl*s may play a similar role in PWD development, essentially at the early stages of PWD.

### 2.5. Detection of RNAi Efficiency

**Figure 6:**
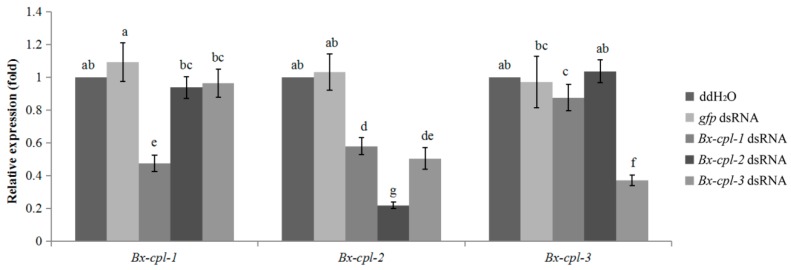
Relative expression levels of *Bx-cpl*s after treatment with *Bx-cpl* double-stranded RNA (dsRNA). The bars indicate standard errors, and different letters indicate significant differences (*p* < 0.05) among treatments: no dsRNA control (ddH_2_O), green fluorescent protein gene (*gfp*) dsRNA control, and each *Bx-cpl* dsRNA.

There were significant differences between each *Bx-cpl* transcript level in nematodes treated with the corresponding double-stranded RNA (dsRNA) and nematodes treated with ddH_2_O or green fluorescent protein gene (*gfp*) dsRNA (controls). The transcripts of *Bx-cpl-1*, *Bx-cpl-2*, and *Bx-cpl-3* decreased (*p* < 0.05) to 47.4, 21.8, and 37.0%, respectively, compared with in the nematodes treated with ddH_2_O (Figure 6). This showed that *Bx-cpls* expression was reduced by soaking the nematodes with the corresponding *Bx-cpl* dsRNA. In addition, dsRNA of *Bx-cpl-1* and *Bx-cpl-3* also targeted *Bx-cpl-2* for degradation.

### 2.6. Feeding and Reproduction of B. xylophilus after RNAi

**Figure 7:**
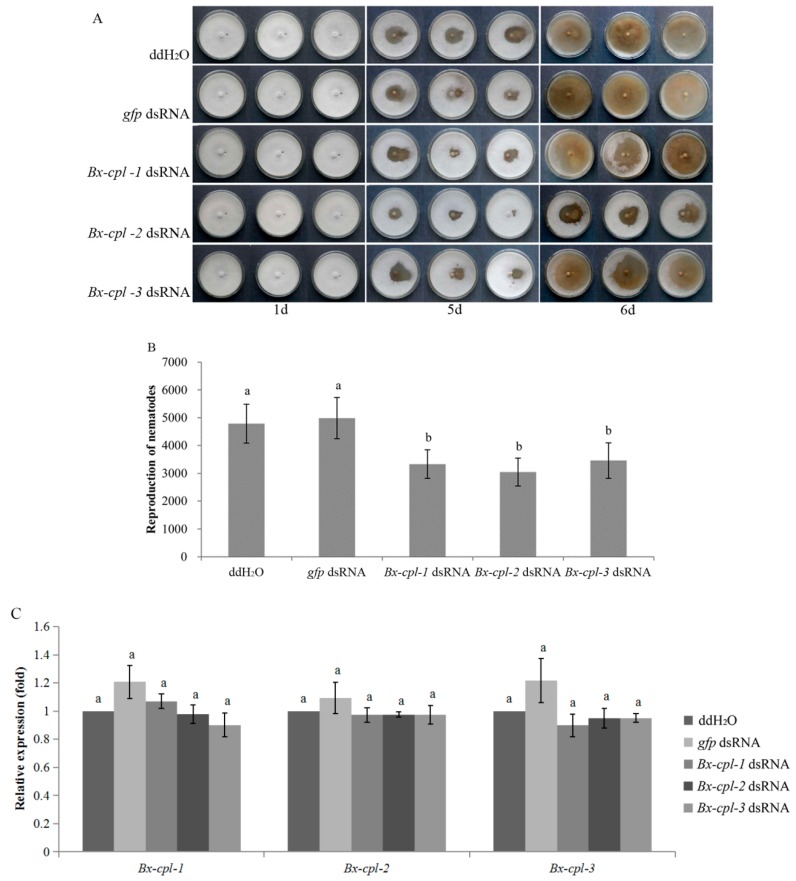
Effects of RNA interference (RNAi) on feeding and reproduction of *B. xylophilus*. RNAi-treated *B. xylophilus* after cultivation on *B. cinerea* (**A**); Total *B. xylophilus* population recovered from *B. cinerea* plates six days after treatment with ddH_2_O and dsRNA (*gfp*, *Bx-cpl-1*, *Bx-cpl-2*, and *Bx-cpl-3*) (**B**); Relative expression levels of *Bx-cpl*s after cultivation on *B. cinerea* for six days (**C**). The bars indicate standard errors between replicates, and different letters indicate significant differences (*p* < 0.05) among treatments.

There was no significant difference in the feeding status between each treatment and the controls until Day 5 of nematode culture on *Botrytis cinerea*. The feeding areas of nematodes treated with ddH_2_O and *gfp* dsRNA were larger than those of nematodes treated with each *Bx-cpl* dsRNA (Figure 7A). At Day 6, the number of nematodes recovered from the culture plates was determined. The PWNs treated with *Bx-cpl* dsRNA were significantly fewer than those treated with ddH_2_O and *gfp* dsRNA (*p* < 0.05), and there was no significant difference (*p* > 0.05) between these two control treatments. Also, no significant differences were found between each *Bx-cpl* dsRNA treatment (Figure 7B). At this time, the expression levels of all three *cpl* genes under each of the five treatments (ddH_2_O and dsRNA (*gfp*, *Bx-cpl-1*, *Bx-cpl-2*, and *Bx-cpl-3*)) were detected. There was no significant difference among all the treatments (*p* > 0.05). These results indicated a deleterious effect of silencing of *Bx-cpl* on the development of *B. xylophilus*, but one which is limited by time.

### 2.7. Pathogenicity of B. xylophilus after RNAi

**Figure 8:**
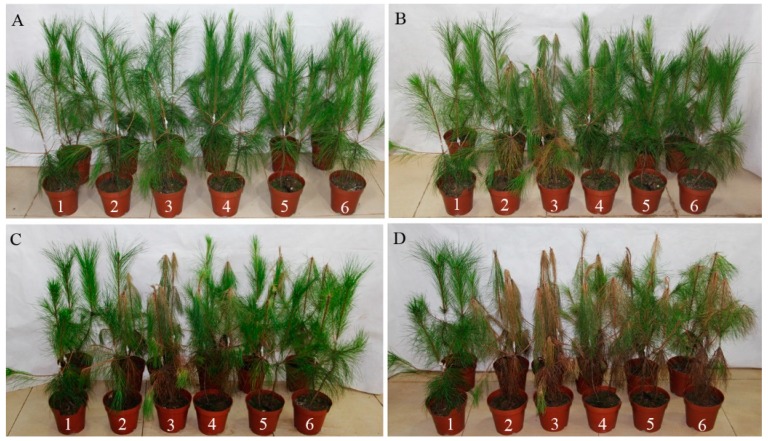
Symptoms in *P. massoniana* after inoculation with nematodes. Symptoms 0 days after inoculation (**A**); Symptoms 8 days after inoculation (**B**); Symptoms 20 days after inoculation (**C**); Symptoms 35 days after inoculation (**D**). Pines inoculated with ddH_2_O (1); *B. xylophilus* soaked in ddH_2_O (2); *B. xylophilus gfp* dsRNA (3); *B. xylophilus Bx-cpl-1* dsRNA (4); *B. xylophilus Bx-cpl-2* dsRNA (5); *B. xylophilus Bx-cpl-3* dsRNA (6).

Five days after inoculation, the pines with nematodes treated with ddH_2_O and *gfp* dsRNA showed clear symptoms. Their infection rates were 25% and 50%, and the disease severity index (DSI) values were 6.25 and 12.25, respectively. At this timepoint, no visible symptoms were registered for pine trees inoculated with nematodes treated with *Bx-cpl* dsRNA. Eight days after inoculation, the pines inoculated with nematodes treated with *Bx-cpl-1* and *Bx-cpl-3* dsRNA showed symptoms with infection rates of 25% and DSI values of 6.25 (Table 1). The pines inoculated with nematodes treated with *Bx-cpl-2* dsRNA presented leaf browning only at the ninth day after inoculation. At Day 20, most of the pines inoculated with PWNs developed symptoms (Figure 8). Thirty-five days after inoculation, the infection rates were all 100%, but the DSI values of the *Bx-cpl-1*, *Bx-cpl-2*, and *Bx-cpl-3* dsRNA treatments (62.5, 50, 56.25) were lower than those of the ddH_2_O and *gfp* dsRNA treatments (93.75, 100). These results showed that the pathogenicity of *B. xylophilus* decreased after treatment with *Bx-cpl* dsRNA.

## 3. Discussion

Cathepsin L-like cysteine proteinase (CPL) is a protease widely distributed in tissues and cells. In many parasitic nematodes, CPL plays an important role in molting, individual development, invasion, feeding on host tissues, and evasion of innate host defenses [28,32,33,34]. However, the roles of CPL in *B. xylophilus* remain unknown.

In this study, the full-length cDNA of three *Bx-cpl*s were cloned, and their amino acid sequences were deduced. Homology analysis showed that the *Bx-cpl-1* deduced protein, Bx-CPL-1, has a close phylogenetic relationship with the CPLs of plant parasitic nematodes such as *D. destructor*, *M. incognita*, and *H. glycines.* Wang et al. [35] analyzed the homology between the deduced protein of a *cpl* sequence from *D. destructor* (ACT35690) and the CPL of *B. xylophilus* (ACH56225.1) and found that the identities were highly similar. The CPL of *M. incognita* also had a high homology with Bx-CPL-1, which plays a crucial role in plant–nematode interaction [30]. The *Bx-cpl-2* and *Bx-cpl-3* deduced proteins (Bx-CPL-2 and Bx-CPL-3) have a close phylogenetic relationship with a CPL of *B. mucronatus* (AID50178.1), which may be related to infection of *B. mucronatus* [36].

ISH enables the investigation of gene expression patterns and gene functions in nematodes [28,37,38]. Hashmi et al. [37] reported that the CPL is widely expressed in the head region, intestines, hypodermal cells, and eggshells of *Caenorhabditis elegans*. However, in plant parasitic nematode *M. incognita*, *Mi-cpl-1* was only expressed in the intestinal cells of *M. incognita* [27,28]. In this study, the localizations of *Bx-cpl-1*, *Bx-cpl-2*, *Bx-cpl-3* were all in the intestine and egg of the female PWN and in the intestine and seminal vesicle of the male PWN. This suggests that the expressions of *Bx-cpl-1*, *Bx-cpl-2*, and *Bx-cpl-3* are similar. The Bx-CPL protein might be involved in the digestive and reproductive processes of *B. xylophilus*.

The CPL could regulate the nematode’s development [39]. In this study, the three *Bx-cpls* were differently expressed in different developmental stages of *B. xylophilus*, and the expressions in eggs were relatively higher than those in juveniles and adults. Hashmi et al. [37] demonstrated that the CPL was essential for the embryogenesis and development of *C. elegans*. This suggests that the *Bx-cpls* might play a role in the development of *B. xylophilus*, especially in embryogenesis.

The relative expression levels of *Bx-cpl-1*, *Bx-cpl-2*, and *Bx-cpl-3* at PWD development stages were also investigated. The transcript levels of the three *Bx-cpls* from *P. massoniana* (except the transcript level of *Bx-cpl-1* at the last stage) were higher than in the nematodes cultured on *B. cinerea.* This characteristic is similar to many pathogenesis-related genes of *B. xylophilus*, such as pectate lyase genes, cytochrome P450 (CYP450) genes, UDP-glucuronosyltrans-ferase (UGT) genes, and ATP-binding cassette (ABC) transporter genes, which were expressed more highly when *B. xylophilus* infected *P. thunbergii* than when it was cultured on *B. cinerea* [40]. Kang et al. [41] constructed subtractive expressed sequence tag (EST) libraries that were specific to the dispersal 4th larval stage (D4S) and the pine-grown propagative mixed stage (PGPS) and found that cysteine protease was highly specific to PGPS compared to D4S. The relative expression levels of *Bx-cpl-1*, *Bx-cpl-2*, and *Bx-cpl-3* were highest at the first stage but declined with the development of the PWD. This suggests that *Bx-cpls* might be associated with the parasitic biology of *B. xylophilus* during its propagation within the host pine tree, especially at the first stage of PWD.

RNAi is a means by which double-stranded RNA (dsRNA) induces sequence-specific post-transcriptional gene silencing [42]. It is a very powerful tool for examining the functions of genes in plant nematodes and other organisms [28,32,43,44]. RNAi-induced gene silencing has previously been achieved in *B. xylophilus* in vitro [21,38,45,46,47]. In this study, the expression levels of *Bx-cpls* treated with *Bx-cpl* dsRNA significantly decreased compared to those of the control groups, indicating that *Bx-cpl* genes could be silenced. In many parasitic nematodes, CPLs have potential roles in invasion and feeding on host tissues, molting, development, and parasitism [27,28,30,37,48,49]. Our results showed that the feeding of PWN weakened, reproduction was reduced, and pathogenicity was lower after silencing *Bx-cpl-1*, *Bx-cpl-2*, or *Bx-cpl-3*, respectively. This suggests that *Bx-cpls* could regulate the nematodes’ reproduction and pathogenicity.

## 4. Materials and Methods

### 4.1. Nematode Culture and Collection

*Bursaphelenchus xylophilus* AMA3 isolated from infected *P. thunbergii* in Maanshan, Anhui, China was provided by the Jiangsu Key Laboratory for Prevention and Management of Invasive Species, Nanjing Forestry University.

The PWNs at different developmental stages were collected according to the method described by Shinya et al. [50] with some modifications. The nematodes were cultured on potato dextrose agar (PDA) covered with *Botrytis cinerea* at 25 °C for 4–5 days and isolated with Baermann funnels. The nematodes were washed three times with distilled water and collected by centrifugation at 3500 rpm for 3 min. Approximately 5000 nematodes were placed in a sterilized plate (3 cm diameter) to lay eggs for 4–6 h at 25 °C under aseptic conditions. The eggs were collected after the nematodes were discarded by sterile water washing. The juveniles, including the second-juveniles (J2), the third-juveniles (J3), and the forth-juveniles (J4), were obtained after the eggs were cultured on a PDA plate containing *B. cinerea* for 30–48 h. The adults, including male and female nematodes, were obtained after the eggs were cultured for 84 h. The nematodes at different developmental stages were identified under a microscope (Leica DM500, Leica Microsystems, Heerbrugg, Switzerland). Then, the nematodes in the same developmental stage were washed three times with distilled water and collected by centrifugation at 3500 rpm for 3 min. The collected nematodes were immediately frozen in liquid nitrogen and stored at −80 °C in a 1.5 mL centrifuge tube for subsequent RNA extraction.

The PWNs at different PWD development stages were collected according to the method described by Ding et al. [51]. The seedlings of *P. massoniana* (2 years old) were disinfected with 75% ethyl alcohol by spraying. Afterwards, 0.5 mL suspensions (about 10,000 mixed-stage nematodes) were pipetted into cutting wounds in *P. massoniana*. Sterile water was used as the control. Then the wounds on *P. massoniana* were sealed by Parafilm. PWNs were collected from three stages based on the corresponding PWD symptoms and inoculation times. In the first stage (F), the tips of the pine needles began to turn brown after pine trees were infected with PWNs. Next, in the middle stage (M), half of the needles on the pine trees turned brown. In the last stage (L), the pine needles were completely brown. PWNs cultured on *B. cinerea* served as a control. The nematodes were extracted with Baermann funnels and washed three times with distilled water. Then, they were collected by centrifugation at 3500 rpm for 3 min, immediately frozen in liquid nitrogen, and stored at −80 °C in a 1.5 mL centrifuge tube for subsequent RNA extraction.

### 4.2. RNA Extraction, PCR Amplification of Bx-cpls, and Phylogenetic Analysis

Total RNA was extracted from the nematodes at each developmental stage and mixed stages using Trizol reagent (Invitrogen, Waltham, MA, USA), measured by ultraviolet absorbance at A260/280 (Eppendorf AG 22331, Hamburg, Germany), and examined by electrophoresis on a 1% agarose gel. The cDNA was synthesized using the TransScript II One-Step gDNA Removal and cDNA Synthesis SuperMix according to the manufacturer’s instructions (TransGen Biotech, Beijing, China). The full-length cDNA sequences of *Bx-cpl-1*, *Bx-cpl-2*, and *Bx-cpl-3* were amplified using the 3′-Full RACE Core Set with the PrimeScript™ RTase kit (TaKaRa Biotechnology, Dalian, China) and 5′-Full RACE Kit with TAP (TaKaRa Biotechnology, Dalian, China). Gene-specific primers were used as follows: *Bx-cpl-1*: GSP1-1 (3′-Full RACE first round of PCR), GSP1-2 (5′-Full RACE first round of PCR), and GSP1-3 (5′-Full RACE second round of PCR); *Bx-cpl-2*: GSP2-1 (3′-Full RACE first round of PCR) and GSP2-2 (5′-Full RACE first round of PCR); *Bx-cpl-3*: GSP3-1 (3′-Full RACE first round of PCR), GSP3-2 (3′-Full RACE second round of PCR), GSP3-3 (5′-Full RACE first round of PCR), and GSP3-4 (5′-Full RACE second round of PCR) (Table 2). They were designed for 3′ and 5′ RACE amplification based on three partially known sequences of *Bx-cpl-1*, *Bx-cpl-2*, and *Bx-cpl-3* which were obtained from the RNA sequencing results [52]. The PCR product was purified, ligated into the vector *pEASY-T1* (TransGen Biotech, Beijing, China), and transformed into *Escherichia coli Trans1-T1* (*E. coli*) competent cells (TransGen Biotech, Beijing, China). The *E. coli* was then incubated overnight at 37 °C on Luria-Bertani (LB) plates containing ampicillin. The positive transformants were analyzed by PCR using primers M13F (-47) and M13R (-48) (Table 2). Once the correct clone was identified, the fresh bacterial suspension was submitted to the Nanjing Genscript sequencing company (Nanjing, China) for sequence analysis. The full-length cDNA sequences of *Bx-cpl-1*, *Bx-cpl-2*, and *Bx-cpl-3* from *B. xylophilus* were submitted to GenBank and assigned the accession numbers MG923677, MG923678, and MG923679. A reference and comparison to the genome data available on WormBase Parasite (BioProject PRJEA64437) were performed using blastn (https://parasite.wormbase.org/Multi/Tools/Blast?db=core) and DNAMAN software (https://www.lynnon.com/index.html). Amino acid sequences of homologous Bx-CPL-1, Bx-CPL-2, and Bx-CPL-3 proteins from other species were obtained from NCBI using blastp. Multiple sequence alignment of deduced protein sequences was carried out with ClustalW in MEGA 7 (https://www.megasoftware.net/) [53]. WAG model with gamma-distributed rates (WAG+G) resulted as the best model by Find Best-fit Substitution Model in MEGA 7. Phylogenetic relationships among the CPLs were inferred by using the Maximum Likelihood (ML) method with a WAG+G model.

### 4.3. In Situ Hybridization (ISH)

The ISH probe templates were generated by PCR based on the full-length cDNA sequences of *Bx-cpl-1*, *Bx-cpl-2*, and *Bx-cpl-3* with the specific primer pairs (Table 2). The DIG-labeled sense RNA probes and antisense RNA probes were synthesized from the PCR products of *Bx-cpls* using the DIG Northern Starter Kit (Roche Diagnostics, Mannheim, Germany) [54]. The nematodes were treated, and hybridizations were performed as described by De Boer et al. [55] using a DIG High Prime DNA Labeling and Detection Starter Kit I (Roche Diagnostics, Mannheim, Germany). For the control group, DIG-labeled sense RNA probes were used. Finally, the nematodes were examined and photographed using a Zeiss Axio Image M2 microscope (Zeiss MicroImaging GmbH, Oberkochen, Germany).

### 4.4. Synthesis of Bx-cpl dsRNA and Interference

Double-stranded RNA (dsRNA) was synthesized using the MEGscript RNAi Kit (Ambion Inc., Austin, TX, USA) with the specific primers containing the T7 promoter (Table 2). The non-endogenous control dsRNA (the green fluorescent protein gene, *gfp*) was synthesized using the specific primers *gfp*-T7-F/*gfp*-R and *gfp*-F/*gfp*-T7-R (Table 2). The RNAi soaking method was performed following the process by Urwin et al. [32]. Approximately 3000 individuals (a mixture of juveniles and adults) of freshly cultured nematodes were soaked in dsRNA solution (800 ng/µL) after being washed with distilled water three times at 3500 rpm for 3 min, and then incubated at 180 rpm for 48 h at 20 °C. The nematodes soaked in the corresponding *gfp* dsRNA and ddH_2_O were used as controls. Each treatment had three replicates. Samples from each treatment were washed thoroughly with ddH_2_O several times after soaking and then used for additional experiments.

### 4.5. The qPCR and Expression Analysis of Bx-cpls

Expressions of *Bx-cpl*s were analyzed using real-time quantitative PCR (qPCR). Total RNA was extracted from 3000 nematodes at each developmental stage and mixed stages using Trizol reagent (Invitrogen, Waltham, MA, USA). The RNA quantity and integrity were checked as previously described. The cDNA was synthesized using TransScript II One-Step gDNA Removal and cDNA Synthesis SuperMix following the manufacturer’s protocol (TransGen Biotech, Beijing, China). Specific primers were designed from the cDNA sequence of target genes using Primer Premier 5.0 (Table 2). The actin gene was amplified as a reference gene using the primers Actin-F/Actin-R (Table 2). The qPCR was performed on ABI Prism 7500 (Applied Biosystems, Foster City, CA, USA) using SYBR Green Master Mix (Vazyme, Nanjing, China). The initial data analysis was performed using ABI Prism 7500 software (https://www.thermofisher.com/cn/zh/home/technical-resources/software-downloads/applied-biosystems-7500-fast-real-time-pcr-system.html) and the 2^−ΔΔCt^ method. All experiments were performed in triplicate with three biological replicates.

### 4.6. Analysis of Reproduction and Pathogenicity of B. xylophilus after RNAi

About 200 nematodes treated with *Bx-cpl* dsRNA were cultured on a PDA plate with *B. cinerea* at 25 °C for 6 days. The ddH_2_O and *gfp* dsRNA were used as controls. Each treatment had three replicates. The feeding of *B. xylophilus* was observed and photographed periodically. Subsequently, the nematodes were washed off the plates using a Baermann funnel. The reproduction rate of the nematodes was counted with an optical microscope (Leica DM500, Leica Microsystems, Heerbrugg, Switzerland). In order to determine the pathogenicity of *B. xylophilus* after RNAi, nearly 2000 nematodes soaked in *Bx-cpl* dsRNA, *gfp* dsRNA, or ddH_2_O without dsRNA were inoculated into each 4-year-old *P. massoniana* seedling. ddH_2_O without nematodes was used as an inoculation control. Each treatment contained four replicates. The inoculated seedlings were placed in a greenhouse. Photographs were taken regularly to record the infection state of the seedlings. PWD symptoms were evaluated and categorized on a scale from 0 to 4 [56]. The categories were as follows: 0 = all needles were green; 1 = 0%–25% of needles were discolored and turned yellow; 2 = 25%–50% of needles turned yellow; 3 = 50%–75% of needles turned yellow; and 4 = 75%–100% of needles turned yellow. The infection rates and the disease severity index (DSI) were calculated using the following formulae:

$$\mathrm{Infection}\;\mathrm{rate}\;=\;\frac{\sum^{\text{​}}\mathrm{Number}\;\mathrm{of}\;\mathrm{infected}\;\mathrm{plants}}{\mathrm{Total}\;\mathrm{number}\;\mathrm{of}\;\mathrm{plants}}\;\times \;100\%,$$

$$\mathrm{DSI}=\frac{\sum^{\text{​}}\mathrm{Number}\;\mathrm{of}\;\mathrm{disease}\;\mathrm{plants}\times \mathrm{symptom}\;\mathrm{stage}}{\mathrm{Total}\;\mathrm{number}\;\mathrm{of}\;\mathrm{plant}\times \mathrm{highest}\;\mathrm{symptom}\;\mathrm{stage}}\times 100.$$

### 4.7. Statistical Analysis

All data are presented as the mean ± standard deviation (Mean ± SD). All parameters were calculated using Microsoft Excel. The statistical significance was determined using SPSS Statistics 17.0 software (IBM China Company Ltd., Beijing, China) with ANOVA and *t*-tests. The level of significance was *p* < 0.05.

## 5. Conclusions

*Bx-cpl-1*, *Bx-cpl-2*, and *Bx-cpl-3* are three cathepsin L-like cysteine proteinase genes of *B. xylophilus* in different genomic locations. Their expressions and functions are similar. They are all involved in the feeding, digestion, development, reproduction, and parasitism of *B. xylophilus*. Silencing of *Bx-cpl* would result in decreasing the feeding ability, number of nematodes, and development of pine wilt disease. These results indicate that cathepsin L-like cysteine proteinase genes play a regulatory role in the development and pathogenicity of the pine wood nematode. This is beneficial to better understanding the molecular mechanisms of development and pathogenicity in *B. xylophilus*.

## Figures and Tables

**Table 1:** Symptoms of *Pinus massoniana* caused by *B. xylophilus* treated with dsRNA.

Treatment	Infection Rates (%)				Disease Severity Index (DSI)			
	5th Day	8th Day	20th Day	35th Day	5th Day	8th Day	20th Day	35th Day
ddH_2_O	25	50	75	100	6.25	25	31.25	93.75
*gfp* dsRNA	50	100	100	100	12.5	37.5	68.75	100
*Bx-cpl-1* dsRNA	0	25	75	100	0	6.25	18.75	62.5
*Bx-cpl-2* dsRNA	0	0	50	100	0	0	12.5	50
*Bx-cpl-3* dsRNA	0	25	50	100	0	6.25	12.5	56.25

**Table 2:** Polymerase chain reaction (PCR) primers.

Name of Primers	Sequence (5′–3′)
**cDNA Cloning of Three Cathepsin L-like Cysteine Proteinase Genes**	
3′ RACE (rapid amplification of cDNA ends) outer primer	TACCGTCGTTCCACTAGTGATTT
3′ RACE inner primer	CGCGGATCCTCCACTAGTGATTTCACTATAGG
GSP (gene specific primer) 1-1	GCAATGGTGGACTTATGGAC
GSP2-1	AATCCAAGAGCCCCGTTATC
GSP3-1	GCACCTACCGAAGCCGATACTA
GSP3-2	CCACTCCAAGACTACCAAGG
5′RACE outer primer	CATGGCTACATGCTGACAGCCTA
5′RACE inner primer	CGCGGATCCACAGCCTACTGATGATCAGTCGATG
GSP1-2	CTTGACGATCCAGTAGTCGC
GSP1-3	CTCGCCATTTGGTCGCATTT
GSP2-2	GGTTCTATCGCCGACATTCT
GSP3-3	AACCAAAGTGTAGCCCCAAT
GSP3-4	TGACCAAAGCGTTGCGAAGT
M13F(−47)	CGCCAGGGTTTTCCCAGTCACGAC
M13R(−48)	AGCGGATAACAATTTCACACAGGA
**Preparation of Template DNA for ISH**	
I-*Bx-cpl-1*-F	CCTTTCGCTGAATACCGTCGTCTTA
I-*Bx-cpl-1*-R	TGATGACTCAAGCCAGCGGATAACT
I-*Bx-cpl-1*-T7-F	TAATACGACTCACTATAGGGCCTTTCGCTGAATACCGTCGTCTTA
I-*Bx-cpl-1*-T7-R	TAATACGACTCACTATAGGGTGATGACTCAAGCCAGCGGATAACT
I-*Bx-cpl-2*-F	GCTGTGGATGTTGCTACGCTTTTGC
I-*Bx-cpl-2*-R	GCTTCTCCGTAGTCCTCTCCCCATT
I-*Bx-cpl-2*-T7-F	TAATACGACTCACTATAGGGGCTGTGGATGTTGCTACGCTTTTGC
I-*Bx-cpl-2*-T7-R	TAATACGACTCACTATAGGGGCTTCTCCGTAGTCCTCTCCCCATT
I-*Bx-cpl-3*-F	ACAGCAGTGCCAAGCCCGCTCAAAT
I-*Bx-cpl-3*-R	GTGCTCGGGCATTGATGATTCCTCC
I-*Bx-cpl-3*-T7-F	TAATACGACTCACTATAGGGACAGCAGTGCCAAGCCCGCTCAAAT
I-*Bx-cpl-3*-T7-R	TAATACGACTCACTATAGGGGTGCTCGGGCATTGATGATTCCTCC
**Preparation of Template DNA for dsRNA**	
*Bx-cpl-1-*T7*-*F	GCCAGTCGTCATCACAAA
*Bx-cpl-1-*R	TGTTCCTCATCGGCTTCT
*Bx-cpl-1-*F	TAATACGACTCACTATAGGGGCCAGTCGTCATCACAAA
*Bx-cpl-1-*T7*-*R	TAATACGACTCACTATAGGGTGTTCCTCATCGGCTTCT
*Bx-cpl-2-*T7*-*F	TAATACGACTCACTATAGGGACTAGATCCCAGCGCCACT
*Bx-cpl-2-*R	AGCCAACAGTCACGACAGC
*Bx-cpl-2-*F	ACTAGATCCCAGCGCCACT
*Bx-cpl-2-*T7*-*R	TAATACGACTCACTATAGGGAGCCAACAGTCACGACAGC
*Bx-cpl-3-*T7*-*F	TAATACGACTCACTATAGGGAGAGCTTCACAGCAGTGCCAAG
*Bx-cpl-3-*R	GTTGAACCTGGTAACTATAGTC
*Bx-cpl-3-*F	GCTTCACAGCAGTGCCAAG
*Bx-cpl-3-*T7*-*R	TAATACGACTCACTATAGGGAGAGTTGAACCTGGTAACTATAGTC
*gfp-*T7*-*F	TAATACGACTCACTATAGGGAGACCATGGCCAACACTTGT
*gfp-*R	AGATAATCCCAGCAGCAGTT
*gfp-*F	AGACCATGGCCAACACTTGT
*gfp-*T7*-*R	TAATACGACTCACTATAGGGAGATAATCCCAGCAGCAGTT
**Real Time PCR**	
q-*Bx-cpl-1-*F	CCAGAAGCCGATGAGGAACA
q-*Bx-cpl-1-*R	CCAGTTTTGTAGAGTTGGAAGC
q-*Bx-cpl-2-*F	AGTCATCGCTGTAATCTGC
q-*Bx-cpl-2-*R	TTGTTGGTGCCATAAGTG
q-*Bx-cpl-3-*F	CTATAACGGAGTCACCTCCAT
q-*Bx-cpl-3-*R	TGCTCTTCACTGAGATCCAGT
Actin-F	GCAACACGGAGTTCGTTGTAGA
Actin-R	GTATCGTCACCAACTGGGATGA

## Supplementary Materials

Supplementary materials are available online http://www.mdpi.com/1422-0067/20/1/215/s1. Figure S1. Full-length cDNA sequences and deduced amino acid sequences of the *Bx-cpls Bx-cpl-1* (A), *Bx-cpl-2* (B), and *Bx-cpl-3* (C). Figure S2: Comparison of *Bx-cpls* gene sequences to genome data of *B. xylophilus*. *Bx-cpl-1* gene (A); *Bx-cpl-2* gene (B); *Bx-cpl-3* gene (C). The intron sequences are underlined.

## References

[1] Shinya R., Morisaka H., Takeuchi Y., Futai K., Ueda M. (2013). Making headway in understanding pine wilt disease: What do we perceive in the postgenomic era?. J. Biosci. Bioeng..

[2] Dwinell L.D. (1993). First report of pinewood nematode (*Bursaphelenchus xylophilus*) in Mexico. Plant Dis..

[3] Mamiya Y. (1988). History of pine wilt disease in Japan. J. Nematol..

[4] Zhang K., Liang J., Yan D.H., Zhang X.Y. (2010). Research advances of pine wood nematode disease in China. World For. Res..

[5] Yi C.K., Byun B.H., Park J.D., Yang S.I., Chang K.H. (1989). First finding of the pine wood nematode, *Bursaphelenchus xylophilus* (Steiner et Buhrer) Nickle and its insect vector in Korea. Res Rep For Res Inst Seoul..

[6] Mota M.M., Braasch H., Bravo M.A., Penas A.C., Burgermeister W., Metge K., Sousa E. (1999). First report of *Bursaphelenchus xylophilus* in Portugal and in Europe. Nematology.

[7] Abelleira A., Picoaga A., Mansilla J.P., Aguin O. (2011). Detection of *Bursaphelenchus xylophilus*, causal agent of pine wilt disease on *Pinus pinaster* in northwestern Spain. Plant Dis..

[8] Khan F.A., Gbadegesin R.A. (1991). On the occurrence of nematode induced pine wilt disease in Nigeria. Pak. J. Nematol..

[9] Kikuchi T., Aikawa T., Kosaka H., Pritchard L., Ogura N., Jones J.T. (2007). Expressed sequence tag (EST) analysis of the pine wood nematode *Bursaphelenchus xylophilus* and *B. mucronatus*. Mol. Biochem. Parasitol..

[10] Kikuchi T., Cotton J.A., Dalzell J.J., Hasegawa K., Kanzaki N., McVeigh P., Takanashi T., Tsai I.J., Assefa S.A., Cock P.J. (2011). Genomic insights into the origin of parasitism in the emerging plant pathogen *Bursaphelenchus xylophilus*. PLoS Pathog..

[11] Shinya R., Morisaka H., Kikuchi T., Takeuchi Y., Ueda M., Futai K. (2013). Secretome Analysis of the pine wood nematode *Bursaphelenchus xylophilus* reveals the tangled roots of parasitism and its potential for molecular mimicry. PLoS ONE.

[12] Tsai I.J., Tanaka R., Kanzaki N., Akiba M., Yokoi T., Espada M., Jones J.T., Kikuchi T. (2016). Transcriptional and morphological changes in the transition from mycetophagous to phytophagous phase in the plant-parasitic nematode *Bursaphelenchus xylophilus*. Mol. Plant Pathol..

[13] Cardoso J., Anjo S., Fonseca L., Egas C., Manadas B., Abrantes I. (2016). *Bursaphelenchus xylophilus* and *B. mucronatus* secretomes: A comparative proteomic analysis. Sci. Rep..

[14] Kikuchi T., Jones J.T., Aikawa T., Kosaka H., Ogura N. (2004). A family of glycosyl hydrolase family 45 cellulases from the pine wood nematode *Bursaphelenchus xylophilus*. FEBS Lett..

[15] Zhang L., Fan Y., Zheng H., Du F., Zhang K.Q., Huang X., Wang L., Zhang M., Niu Q. (2013). Isolation and characterization of a novel endoglucanase from a *Bursaphelenchus xylophilus* metagenomic library. PLoS ONE.

[16] Kikuchi T., Shibuya H., Aikawa T., Jones J.T. (2006). Cloning and characterization of pectate lyases expressed in the esophageal gland of the pine wood nematode *Bursaphelenchus xylophilus*. Mol. Plant Microbe Interact..

[17] Lee D.W., Kang J.S., Jung C.S., Han H.R., Moon Y.S., Park S.J., Lee S.H., Koh Y.H. (2013). Identification and biochemical analysis of a novel pectate lyase 3 gene in *Bursaphelenchus xylophilus*. J. Asia-Pac. Entomol..

[18] Kikuchi T., Li H.M., Karim N., Kennedy M.W., Moens M., Jones J.T. (2009). Identification of putative expansin-like genes from the pine wood nematode, *Bursaphelenchus xylophilus*, and evolution of the expansin gene family within the nematoda. Nematology.

[19] Kim Y.H., Kim A.Y., Choi B.H., Han H.R., Koh Y.H. (2017). ExpansinB3 as a marker for detecting pine wood nematode-infected pine trees. J. Asia-Pac. Entomol..

[20] Lin S.F., Jian H., Zhao H.J., Yang D., Liu Q. (2011). Cloning and characterization of a venom allergen-like protein gene cluster from the pinewood nematode *Bursaphelenchus xylophilus*. Exp. Parasitol..

[21] Xu X.L., Wu X.Q., Ye J.R., Huang L. (2015). Molecular characterization and functional analysis of three pathogenesis-related cytochrome P450 genes from *Bursaphelenchus xylophilus* (Tylenchida, Aphelenchoidoidea). Int. J. Mol. Sci..

[22] Rhoads M.L., Fetterer R.H. (1997). Extracellular matrix: A tool for defining the extracorporeal function of parasite proteases. Parasitol. Today.

[23] Malagón D., Benítez R., Kašný M., Adroher F.J., Erzinger G.S. (2013). Peptidases in parasitic nematodes. A review. Parasites: Ecology, Diseases and Management.

[24] Sajid M., McKerrow J.H. (2002). Cysteine proteases of parasitic organisms. Mol. Biochem. Parasitol..

[25] Britton C., Murray L. (2002). A cathepsin L protease essential for *Caenorhabditis elegans* embryogenesis is functionally conserved in parasitic nematodes. Mol. Biochem. Parasitol..

[26] Urwin P.E., Lilley C.J., McPherson M.J., Atkinson H.J. (1997). Characterization of two cDNAs encoding cysteine proteinases from the soybean cyst nematode *Heterodera glycines*. Parasitology.

[27] Neveu C., Abad P., Castagnone-Sereno P. (2003). Molecular cloning and characterization of an intestinal cathepsin L protease from the plant-parasitic nematode *Meloidogyne incognita*. Physiol. Mol. Plant Pathol..

[28] Shingles J., Lilley C.J., Atkinson H.J., Urwin P.E. (2007). *Meloidogyne incognita*: Molecular and biochemical characterisation of a cathepsin L cysteine proteinase and the effect on parasitism following RNAi. Exp. Parasitol..

[29] Neveu C., Jaubert S., Abad P., Castagnone-Sereno P. (2003). A set of genes differentially expressed between avirulent and virulent *Meloidogyne incognita* near-isogenic lines encode secreted proteins. Mol. Plant Microbe Interact..

[30] Dutta T.K., Papolu P.K., Banakar P., Choudhary D., Sirohi A., Rao U. (2015). Tomato transgenic plants expressing hairpin construct of a nematode protease gene conferred enhanced resistance to root-knot nematodes. Front. Microbiol..

[31] Duarte A., Maleita C., Tiago I., Curtis R., Abrantes I. (2016). Molecular characterization of putative parasitism genes in the plant-parasitic nematode *Meloidogyne hispanica*. J. Helminthol..

[32] Urwin P.E., Lilley C.J., Atkinson H.J. (2002). Ingestion of double-stranded RNA by preparasitic juvenile cyst nematodes leads to RNA interference. Mol. Plant Microbe Interact..

[33] Dalton J.P., Neill S.O., Stack C., Collins P., Walshe A., Sekiya M., Doyle S., Mulcahy G., Hoyle D., Khaznadji E. (2003). *Fasciola hepatica* cathepsin L-like proteases: Biology, function, and potential in the development of first generation liver fluke vaccines. Int. J. Parasitol..

[34] Corvo I., Cancela M., Cappetta M., Pi-Denis N., Tort J.F., Roche L. (2009). The major cathepsin L secreted by the invasive juvenile *Fasciola hepatica* prefers proline in the S2 subsite and can cleave collagen. Mol. Biochem. Parasitol..

[35] Wang G.F., Peng D.L., Sun J.H., Huang W.K., Peng H., Long H.B. (2011). Cloning and sequence analysis of a new cathepsin L-like cysteine proteinase gene from *Ditylenchus destructor*. Chin. J. Biotechnol..

[36] Pan Y.Y., Huang L., Wu X.Q. (2015). Bioinformatic and expression analysis of a cathepsin gene Bmcath1 in *Bursaphelechus mucronatus*. J. Nanjing For. Univ..

[37] Hashmi S., Britton C., Liu J., Guiliano D.B., Oksov Y., Lustigman S. (2002). Cathepsin L is essential for embryogenesis and development of *Caenorhabditis elegans*. J. Biol. Chem..

[38] Deng L.N., Wu X.Q., Ye J.R., Xue Q. (2016). Identification of autophagy in the pine wood nematode *Bursaphelenchus xylophilus* and the molecular characterization and functional analysis of two novel autophagy-related genes, BxATG1 and BxATG8. Int. J. Mol. Sci..

[39] Rhoads M.L., Fetterer R.H. (1995). Developmentally regulated secretion of cathepsin L-like cysteine proteases by *Haemonchus contortus*. J. Parasitol..

[40] Qiu X.W., Wu X.Q., Huang L., Tian M.Q., Ye J.R. (2013). Specifically expressed genes of the nematode *Bursaphelenchus xylophilus* involved with early interactions with pine trees. PLoS ONE.

[41] Kang J.S., Lee H., Moon I.S., Lee Y., Koh Y.H., Je Y.H., Lim K.J., Lee S.H. (2009). Construction and characterization of subtractive stage-specific expressed sequence tag (EST) libraries of the pinewood nematode *Bursaphelenchus xylophilus*. Genomics.

[42] Fire A., Xu S., Montgomery M.K., Kostas S.A., Driver S.E., Mello C.C. (1998). Potent and specific genetic interference by double-stranded RNA in *Caenorhabditis elegans*. Nature.

[43] Rosso M.N., Dubrana M.P., Cimbolini N., Jaubert S., Abad P. (2005). Application of RNA interference to root-knot nematode genes encoding esophageal gland proteins. Mol. Plant Microbe Interact..

[44] Li Y., Xie H., Xu C.L., Li D.L., Zhang C. (2010). RNAi effect of cathepsin B gene on reproduction of *Radopholus similis*. Sci. Agric. Sin..

[45] Li X.D., Zhuo K., Luo M., Sun L., Liao J. (2011). Molecular cloning and characterization of a calreticulin cDNA from the pinewood nematode *Bursaphelenchus xylophilus*. Exp. Parasitol..

[46] Wang X.R., Cheng X., Li Y.D., Zhang J.A., Zhang Z.F., Wu H.R. (2012). Cloning arginine kinase gene and its RNAi in *Bursaphelenchus xylophilus* causing pine wilt disease. Eur. J. Plant Pathol..

[47] Cardoso J.M.S., Fonseca L., Gomes P., Egas C., Abrantes I. (2015). Molecular characterization and functional analysis of a calponin gene from the pinewood nematode. Forest Pathol..

[48] Koiwa H., Shade R.E., Zhu-Salzman K., D’Urzo M.P., Murdock L.L., Bressan R.A., Hasegawa P.M. (2000). A plant defensive cystatin (soya cystatin) targets cathepsin L-like digestive cysteine proteinases (DvCALs) in the larval midgut of western corn rootworm (*Diabrotica virgifera*). FEBS Lett..

[49] Lustigman S., Zhang J., Liu J., Oksov Y., Hashmi S. (2004). RNA interference targeting cathepsin L and Z-like cysteine proteases of *Onchocerca volvulus* confirmed their essential function during L3 molting. Mol. Biochem. Parasitol..

[50] Shinya R., Takeuchi Y., Futai K. (2009). A technique for separating the developmental stages of the propagative form of the pine wood nematode, *Bursaphelenchus xylophilus*. Nematology.

[51] Ding X.L., Ye J.R., Wu X.Q., Huang L., Zhu L.H., Lin S.X. (2015). Deep sequencing analyses of pine wood nematode *Bursaphelenchus xylophilus* microRNAs reveal distinct miRNA expression patterns during the pathological process of pine wilt disease. Gene..

[52] He L.X., Wu X.Q., Xue Q., Qiu X.W. (2016). Effects of endobacterium (*Stenotrophomonas maltophilia*) on pathogenesis-related gene expression of pine wood nematode (*Bursaphelenchus xylophilus*) and pine wilt disease. Int. J. Mol. Sci..

[53] Kumar S., Stecher G., Tamura K. (2016). MEGA7: Molecular Evolutionary Genetics Analysis version 7.0 for bigger datasets. Mol. Biol. Evol..

[54] Regina W., Corinna W., Alexandra F., Jarutat T., Astrid H., Tobias B., William D., Barbara R. A Method for High Quality Digoxigenin-Labeled RNA Probes for in Situ Hybridization. http://www.ebiotrade.com/custom/upload/140506/1.pdf.

[55] De Boer J.M., Yan Y., Smant G., Davis E.L., Baum T.J. (1998). In-situ hybridization to messenger RNA in *Heterodera glycines*. J. Nematol..

[56] Yu L.Z., Wu X.Q., Ye J.R., Zhang S.N., Wang C. (2012). NOS-like-mediated nitric oxide is involved in *Pinus thunbergii* response to the invasion of *Bursaphelenchus xylophilus*. Plant Cell Rep..

