# Supplementary material for: Cathepsin L-like Cysteine Proteinase Genes Are Associated with the Development and Pathogenicity of Pine Wood Nematode, Bursaphelenchus xylophilus
Source: Int J Mol Sci. 2019 Jan 8;20(1):215. doi: 10.3390/ijms20010215 (PMC6337200; doi:10.3390/ijms20010215)
Supplement: Supplementary file 1 [file ijms-20-00215-s001.zip › Supplementary File/ijms-372744 with track changes.docx]

Article

Cathepsin L-like cysteine proteinase genes are associated with the development and pathogenicity of pine wood nematode, *Bursaphelenchus xylophilus*

Qi Xue^1, 2^, Xiao-Qin Wu^1, 2^*, Wan-Jun Zhang^1, 2^, Li-Na Deng^3^, Miao-Miao Wu^1, 2^

^1^ Co-Innovation Center for Sustainable Forestry in Southern China, College of Forestry, Nanjing Forestry University, Nanjing, Jiangsu 210037, China

^2^ Jiangsu Key Laboratory for Prevention and Management of Invasive Species, Nanjing Forestry University, Nanjing, Jiangsu 210037, China

^3^ Yancheng Institute of Technology, School of Ocean and Biological Engineering, Yancheng, Jiangsu 224051, China

***** Correspondence: [xqwu_njfu](mailto:xqwu_njfu)@163.com; Tel.: +86-25-8542-7427

Received: 29 September 2018; Accepted: date; Published: date

**Abstract:** The pine wood nematode (PWN), Bursaphelenchus xylophilus, is the pathogen of pine wilt disease (PWD) resulting in huge losses in pine forests. However, its pathogenic mechanism remains unclear. The cathepsin L-like cysteine proteinase (CPL) genes are multifunctional genes related to the parasitic abilities of plant-parasitic nematodes, but their functions in PWN remain unclear. We cloned three *cpl* genes of PWN (*Bx-cpls*) by rapid-amplification of cDNA ends (RACE), and analyzed their characteristics with bioinformatic methods. The tissue specificity of *Bx-cpl* was researched by in situ mRNA hybridization (ISH). The function of *Bx-cpl*s in development and pathogenicity were investigated using qPCR and RNA interference (RNAi). The results showed that the full-length cDNAs of *Bx-cpl-1*, *Bx-cpl-2*, and *Bx-cpl-3* were 1163 bp, 1305 bp and 1302 bp, respectively. *Bx-cpl*s could accumulate specifically in the egg, intestine and genital system of PWN. During different developmental stages of PWN, the expression of *Bx-cpl*s in the egg stage were highest. After infection, the expressions of *Bx-cpl*s increased, and reached highest at the initial stage of PWD, then declined gradually. The silencing of *Bx-cpl* could reduce feeding, reproduction and pathogenicity of PWN. These results revealed that *Bx-cpl*s play multiple roles in the development and pathogenic process of PWN.

**Keywords:** Bursaphelenchus xylophilus; cathepsin L; gene expression; development; pathogenicity

1. Introduction

The pine wood nematode (PWN), *Bursaphelenchus xylophilus* (Steiner & Buhrer) Nickle, is the causal agent of pine wilt disease (PWD). It has been detected in North America (USA, Canada, and Mexico) [1, 2], East Asia (Japan, China, and Korea) [3, 4, 5], Europe (Portugal and Spain) [6, 7], and Nigeria [8]. The disease has been unquestionably a major threat to forest ecosystems worldwide and has caused great losses in China. However, the pathogenic mechanism of *B. xylophilus* remains unclear.

With the development of biotechnology, ESTs, genome, transcriptome and secretome of *B. xylophilus* have been analyzed highlighting several groups of genes putatively related to its pathogenicity [9-13]. Cellulase genes [14, 15], pectatelyase genes [16, 17], expansin-like genes [18, 19], the venom allergen-like protein gene [20], and cytochrome P450 genes [21] have been studied and identified as pathogenesis-related genes. The functions of other putative pathogenesis-related genes of *B. xylophilus* still need to be identified*.*

It is believed that peptidases are essential for the parasite development and in the most critical situation of parasite-host interactions. Peptidases comprise a large class of hydrolytic enzymes in parasites [22]. Of these, the cysteine peptidases are the class that covers virtually all functions that involve peptidases in parasitic helminths (including trematodes, cestodes, and nematode parasites) [23]. Cathepsin L is a type of cysteine peptidase belonging to the papain family, and has been comprehensively studied in many parasitic helminths [24]. In free-living and parasite nematodes of humans and animals, the cathepsin L proteinases are involved in pivotal functions, such as tissue penetration, nutrition, immune evasion and eggshell formation, although little is known of their precise functions [25]. As with animal parasite counterparts, nematodes that infect plants may require proteinases for egg hatching, larval molting, tissue penetration and feeding. Urwin et al. [26] were the first team to clone a cathepsin L-like proteinase (CPL) from *Heterodera glycines*. To date, a number of *cpl* genes from plant parasitic nematodes including *Bursaphelenchus*, *Globodera*, *Heterodera*, *Meloidogyne*, and *Rotylenchulus* have been cloned, but their functions are seldom reported formally. The *cpl* gene of *Meloidogyne incognita* (*Mi-cpl-1*) encodes a digestive enzyme which is consistent with feeding [27, 28]. In addition, *Mi-cpl-1* can affect *M. incognita* development and play a crucial role in plant-nematode interactions [28-30]. The *cpl* gene in *M. hispanica* is also identified and characterized as a parasitism gene [31]. In *B. xylophilus*, two *cpl* genes (ACH69776.1, ACH56225.1) have been cloned, but their functions have not yet been investigated. In this study, the full-length cDNA of three novel *cpl* genes, *Bx-cpl-1*, *Bx-cpl-2*, and *Bx-cpl-3,* were cloned using 3’ and 5’ rapid-amplification of cDNA ends (RACE). The expressions of *Bx-cpls* in *B. xylophilus* at different developmental and pathogenic stages associated with PWD were analyzed by qPCR. The roles of *Bx-cpls* in the reproduction and pathogenicity were verified through RNA interference (RNAi). These results provide useful information to better understand the functions of *cpl*s in *B. xylophilus*, and elucidate the molecular pathogenic mechanism.

2. Results

2.1. Cloning and sequence analysis of three cathepsin L-like cysteine proteinase genes from B. xylophilus

The complete nucleotide sequence of *Bx-cpl-1* had 1163 bp (Figure 1), including a 18 bp 5’ untranslated region (UTR), a 1074 bp open reading frame (ORF), and a 71 bp 3’ UTR. It encoded a protein of 357 amino acid residues (S1A Figure). The full-length cDNA of *Bx-cpl-2* had 1305 bp (Figure 1), comprising a 48 bp 5’ UTR, an 1185 bp ORF encoding 394 amino acid residues, and a 72 bp 3’ UTR (S1B Figure). The full-length cDNA of *Bx-cpl-3* was 1302 bp (Figure 1), including a 51 bp 5’ UTR, a 63 bp 3’ UTR, and an 1188 bp ORF encoding for 395 amino acids (S1C Figure). Compared to the genome data available on WormBase Parasite (BioProject PRJEA64437), the genomic locations of *Bx-cpl-1*, *Bx-cpl-2* and *Bx-cpl-3* were at scaffold01141 191468 to 192800 with three introns, scaffold00813 265318 to 266698 with two introns, and scaffold01147 920147 to 921523 with two introns, respectively (S2 Figure).

**
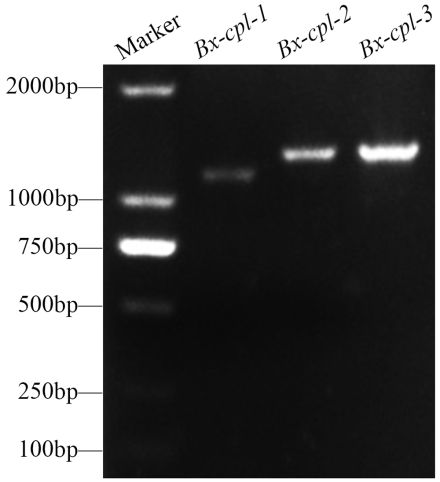
**

**Figure 1.** Bands of *Bx-cpls* full length cDNA sequences after gel eletrophoresis.

The results of Blastp showed that some CPL proteins have a relatively high level of identity with the CPLs of *B.* *xylophilus*. On this basis, amino acid sequences from Nematode and Protozoa, which showed relatively high homology with the predicted amino acid sequences from *B.* *xylophilus* were selected and downloaded from NCBI. The phylogenetic tree was constructed by the maximum likelihood method with WAG+G model based on the amino acid sequences of CPL proteins (Figure. 2). Three CPL proteins of *B.* *xylophilus* were divided into two nematode groups. Bx-CPL-1 was closely related to plant parasitic nematodes, *Ditylenchus destructor, Meloidogyne incognita, Heterodera glycines, Globodera pallida,* especially *B.* *xylophilus* (ACH56225.1). However, Bx-CPL-2 and Bx-CPL-3 were highly linked to *B.* *mucronatus* (AID50178.1) and even the CPLs in Protozoa, rather than the other CPLs in Nematode (Figure. 2).


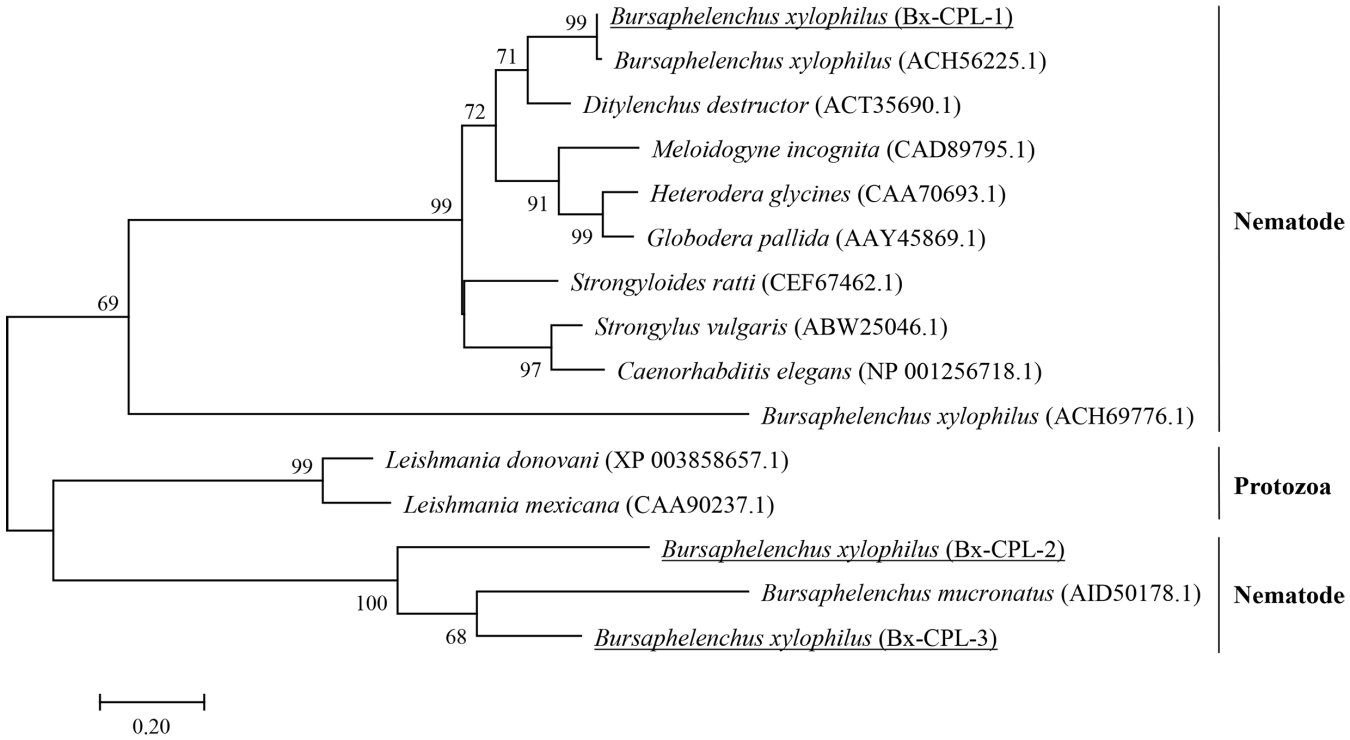


**Figure 2.** Phylogenetic relationships of CPLs. The phylogram was constructed based on amino acid sequences to determine the evolutionary relationships among 15 CPL proteins from diﬀerent species using MEGA 7. The numbers below the branches indicate the bootstrap values, which were calculated from 1000 replicates. The GenBank accession numbers of the sequences are in brackets. *B.* *xylophilus* CPLs were underlined. Distance scale = 0.2.

2.2. Localization of Bx-cpl in B. xylophilus

In situ hybridization (ISH) was used to analyze the tissue specificity of *Bx-cpl* transcription. The localizations of three *Bx-cpls* were similar. The digoxigenin (DIG)-labeled antisense RNA probe of *Bx-cpl* generated clear signals in the intestine and egg of females (Figure A, B), and intestine and seminal vesicle of male of *B. xylophilus* (Figure C)*.* No signals were observed in the control group with the sense probes.


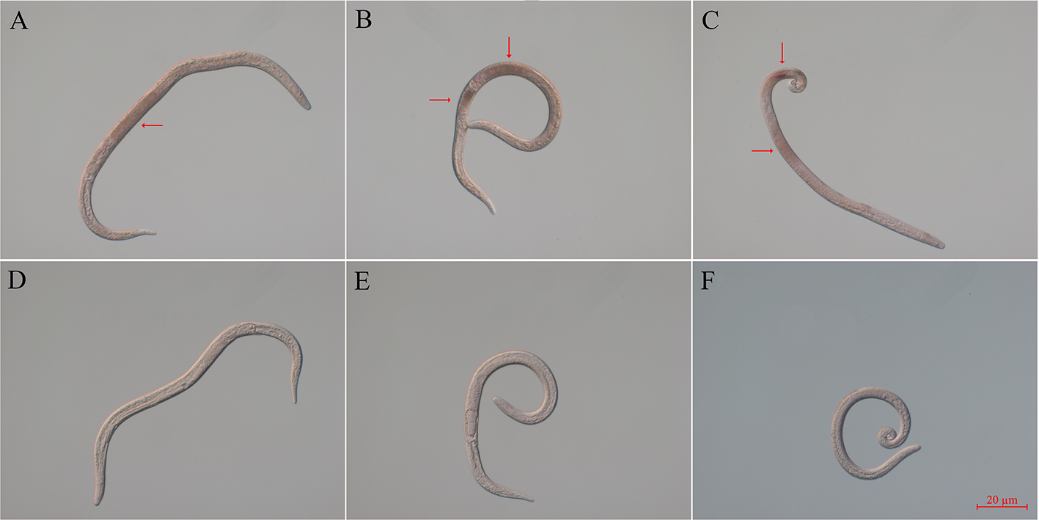


**Figure 3.** Localizations of *Bx-cpl*s mRNA by in situ hybridization (ISH). *Bx-cpl* was expressed in PWN’s intestine of female (**A**), intestine and egg of female (**B**), intestine and seminal vesicle of male (**C**). The control groups were no signals (**D**) (**E**) (**F**). The scale bars =20 µm.

2.3. Expression of Bx-cpl at PWN’s developmental stages

All *Bx-cpl*s showed relatively high transcript levels in the egg stage. The *Bx-cpl-1* expression was significantly lower in adults than in juveniles (*p* < 0.05) and *Bx-cpl-2* expression was the opposite. There was no significant difference of *Bx-cpl-3* expression between juveniles and adults (*p* > 0.05) (Figure 4).


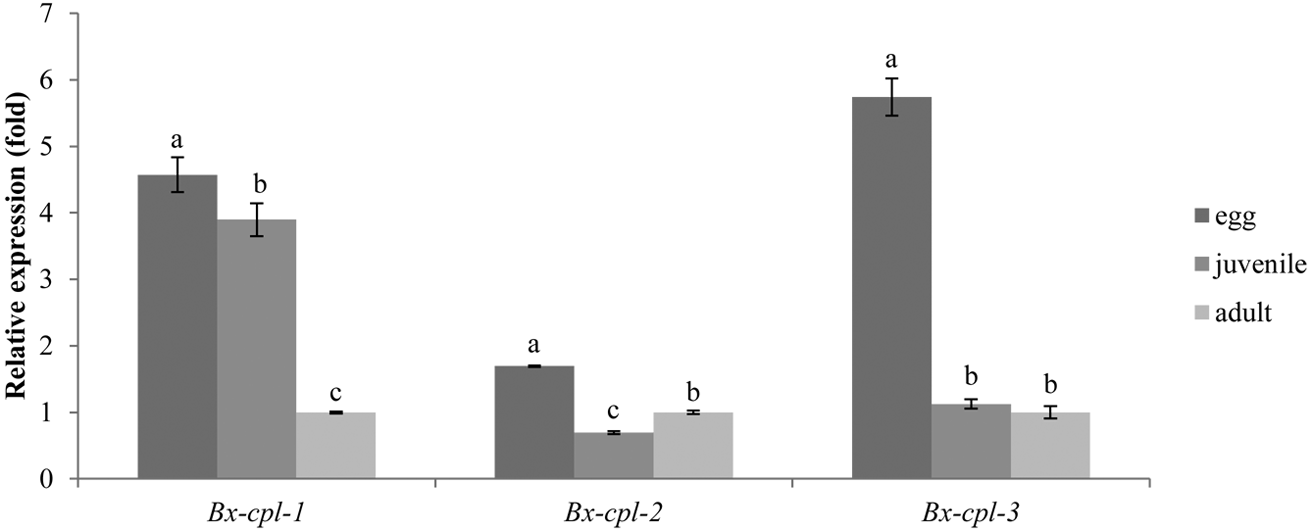


**Figure 4.** Relative expression levels of *Bx-cpl*s at different developmental stages of *B. xylophilus*. The bars indicate standard errors, and diﬀerent letters indicate significant diﬀerences (*p* < 0.05) among the different nematode stages (egg, juvenile and adult).

2.4. Expression of Bx-cpl at PWD development stages

After infection of pine seedlings with *B. xylophilus*, all three *Bx-cpl*s were found upregulated and reached the highest expression level at the first stage of PWD. Then their expression declined and downed to the lowest level at the late stage (Figure 5). These results indicate that three *Bx-cpl*s may play a similar role in PWD development, essentially at the early stage of PWD.


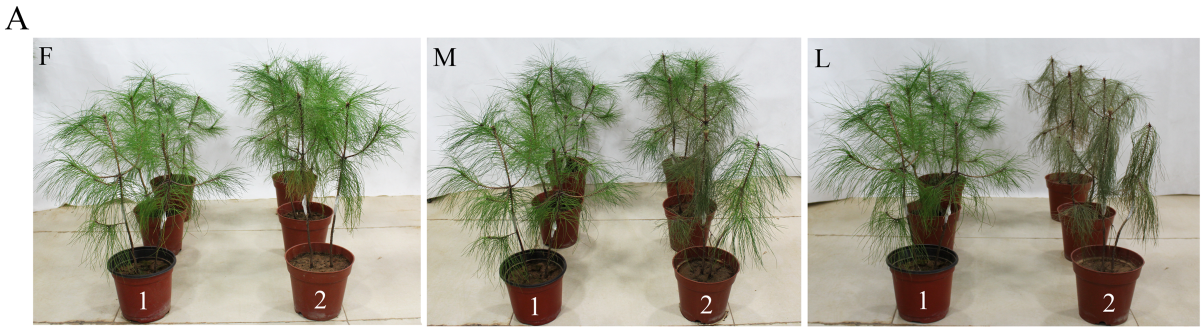





**Figure 5.** Symptoms in *P. massoniana* after inoculation with nematodes (**A**). first stage of PWD (F), middle stage of PWD (M) and last stage of PWD (L). Pines inoculated with ddH_2_O (1); Pines inoculated with *B. xylophilus* (2). Relative expression levels of *Bx-cpl*s at PWD development stages (**B**). The bars indicate standard errors, and diﬀerent letters indicate significant diﬀerences (*p* < 0.05).

2.5. Detection of RNAi Efficiency

There were significant differences between each *Bx-cpl* transcript level in nematodes treated with the corresponding dsRNA and nematodes treated with ddH_2_O or *gfp* dsRNA (controls). The transcripts of *Bx-cpl-1*, *Bx-cpl-2* and *Bx-cpl-3* decreased (*p* < 0.05) to 47.4, 21.8, and 37.0 %, respectively, compared to the nematodes treated with ddH_2_O (Figure. 6). This showed that *Bx-cpls* expression was reduced by soaking the nematodes with the corresponding *Bx-cpl* dsRNA. In addition, dsRNA of *Bx-cpl-1* and *Bx-cpl-3* also targeted *Bx-cpl-2* for degradation.


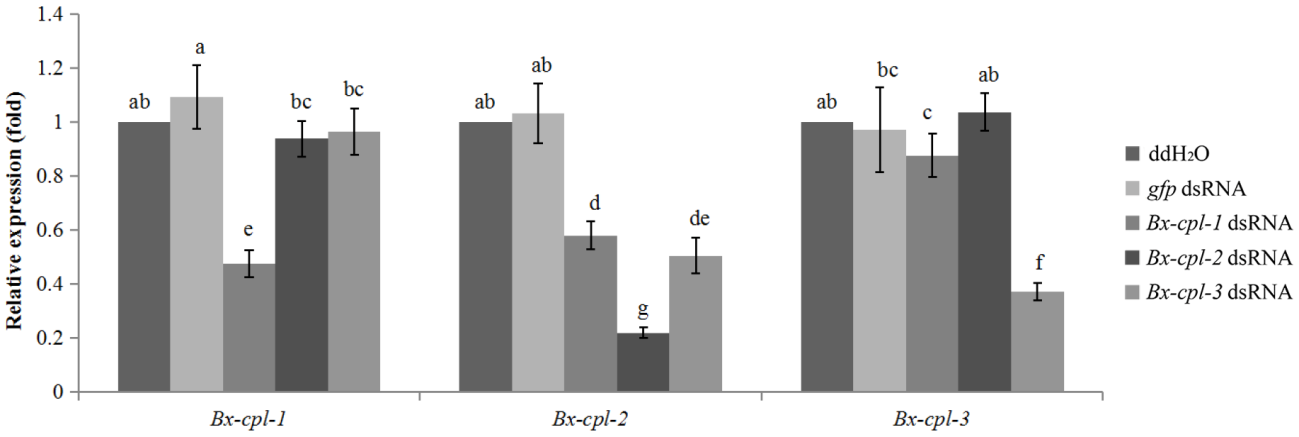


**Figure 6.** Relative expression levels of *Bx-cpl*s after treated with *Bx-cpl* dsRNA. The bars indicate standard errors, and diﬀerent letters indicate significant diﬀerences (*p* < 0.05) among treatments: no dsRNA control (ddH_2_O), *gfp* dsRNA control and each *Bx-cpl* dsRNA.

2.6. Feeding and reproduction of B. xylophilus after RNAi

There was no significant difference in the feeding status between each treatment and the controls until day 5 of nematodes culture on *Botrytis cinerea*. The feeding area of nematodes treated with ddH_2_O and *gfp* dsRNA were larger than those of nematodes treated with each *Bx-cpl* dsRNA (Figure 7A). At day 6, the number of nematodes recovered from the culture plates was determined. The PWNs treated with *Bx-cpl* dsRNA was significantly fewer than those treated with ddH_2_O and *gfp* dsRNA (*p* < 0.05) and there was no significant difference (*p* > 0.05) between these two control treatments. Also no significant differences were found between each *Bx-cpl* dsRNA treatment (Figure 7B). At this time, the expression levels of all three *cpl* genes under each treatment of five treatments (ddH_2_O and dsRNA (*gfp*, *Bx-cpl-1*, *Bx-cpl-2* and *Bx-cpl-3*)) were detected. There was no significant difference among all the treatments (*p* > 0.05). This results indicated a deleterious effect of silencing of *Bx-cpl* on the development of *B. xylophilus*, but limited by time.

**
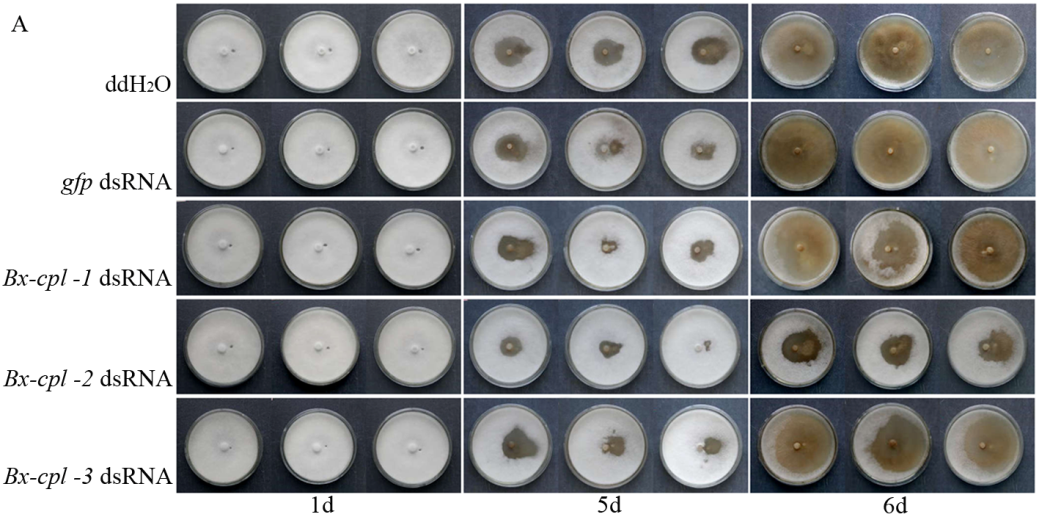
**

**

**

**
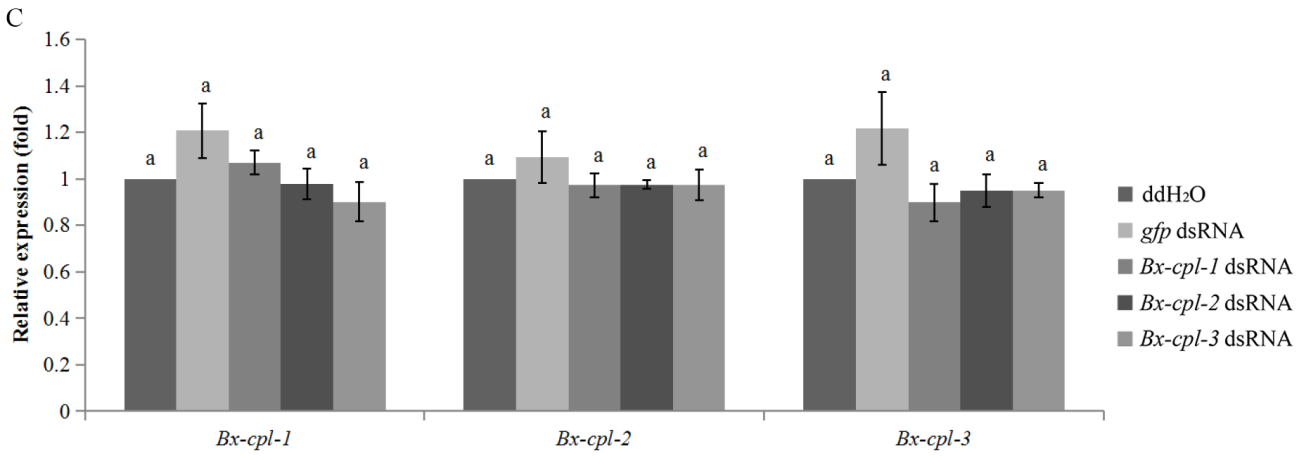
**

**Figure 7.** Effects of RNAi on feeding and reproduction of *B. xylophilus*. RNAi-treated B. xylophilus after cultivation on B. cinerea (**A**); Total number of B. xylophilus recovered from B. cinerea plates six days after treatment with ddH_2_O and dsRNA (*gfp*, *Bx-cpl-1*, *Bx-cpl-2* and *Bx-cpl-3*) (**B**); Relative expression levels of *Bx-cpl*s after cultivation on B. cinerea six days (**C**). The bars indicate standard errors between replicates, and diﬀerent letters indicate significant diﬀerences (*p* < 0.05) among treatments.

2.7. Pathogenicity of B. xylophilus after RNAi

Five days after inoculation, the pines with nematodes treated with ddH_2_O and *gfp* dsRNA showed clear symptoms. Their infection rates were 25% and 50% and the disease severity index (DSI) were 6.25 and 12.25, respectively. At this time point no visible symptoms were registered for pine trees inoculated with nematodes treated with *Bx-cpl* dsRNA. Eight days after inoculation, the pines inoculated with nematodes treated with *Bx-cpl-1* and *Bx-cpl-3* dsRNA showed symptoms with infection rates of 25% and DSI of 6.25 (Table 2). The pines inoculated with nematodes treated with *Bx-cpl-2* dsRNA presented leaf browning only at the 9th day after inoculation. At the day 20, most pines inoculated with PWNs developed symptoms (Figure 8). Thirty five days after inoculation, the infection rates were all 100%, but the DSIs of *Bx-cpl-1*, *Bx-cpl-2* and *Bx-cpl-3* dsRNA treatments (62.5, 50, 56.25) were the lower than those of ddH_2_O and *gfp* dsRNA treatments (93.75, 100). These results showed that the pathogenicity of *B. xylophilus* decreased after treated with *Bx-cpl* dsRNA.

**Table 2.** Symptoms of *Pinus massoniana* caused by *B. xylophilus* treated with dsRNA.

| Treatment | Infection rates (%) | | | |  | Disease Severity Index (DSI) | | | |
| --- | --- | --- | --- | --- | --- | --- | --- | --- | --- |
|  | 5^th^ day | 8^th^ day | 20^th^ day | 35^th^ day |  | 5^th^ day | 8^th^ day | 20^th^ day | 35^th^ day |
| ddH_2_O | 25 | 50 | 75 | 100 |  | 6.25 | 25 | 31.25 | 93.75 |
| *gfp* dsRNA | 50 | 100 | 100 | 100 |  | 12.5 | 37.5 | 68.75 | 100 |
| *Bx-cpl-1* dsRNA | 0 | 25 | 75 | 100 |  | 0 | 6.25 | 18.75 | 62.5 |
| *Bx-cpl-2* dsRNA | 0 | 0 | 50 | 100 |  | 0 | 0 | 12.5 | 50 |
| *Bx-cpl-3* dsRNA | 0 | 25 | 50 | 100 |  | 0 | 6.25 | 12.5 | 56.25 |

**
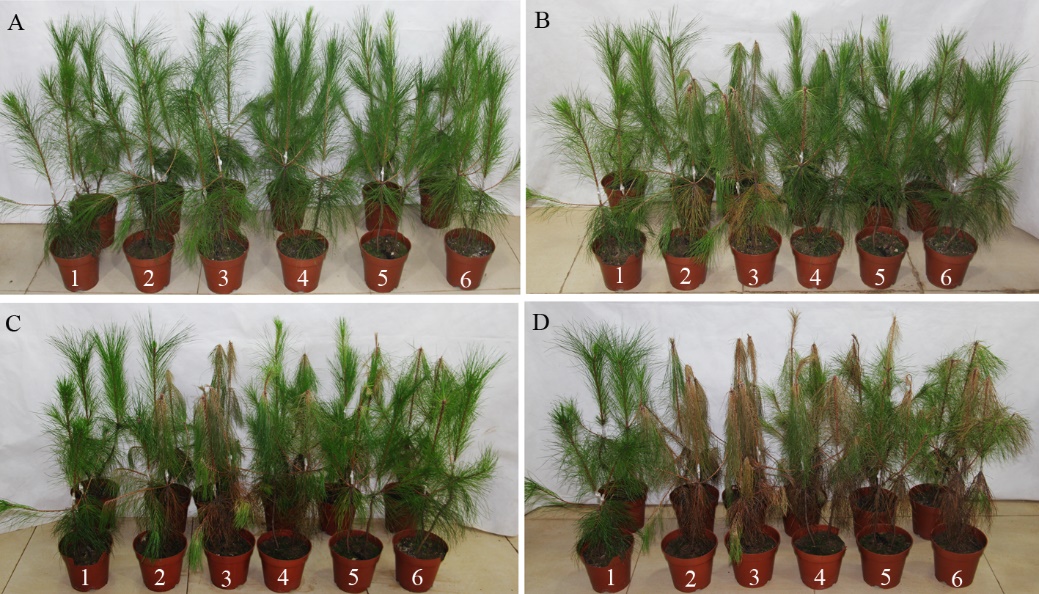
**

**Figure 8.** Symptoms in *P. massoniana* after inoculation with nematodes. Symptoms 0 days after inoculation (**A**); Symptoms 8 days after inoculation (**B**); Symptoms 20 days after inoculation (**C**); Symptoms 35 days after inoculation (**D**). Pines inoculated with ddH_2_O (1); *B. xylophilus* soaked in ddH_2_O (2); *B. xylophilus* *gfp* dsRNA (3); *B. xylophilus* *Bx-cpl-1* dsRNA (4); *B. xylophilus* *Bx-cpl-2* dsRNA (5); *B. xylophilus* *Bx-cpl-3* dsRNA (6).

3. Discussion

Cathepsin L-like cysteine proteinase (CPL) is a protease widely distributed in tissues and cells. In many parasitic nematodes, CPL plays an important role in molting, individual development, invasion, feeding on host tissues, and evasion of innate host defenses [28, 32-34]. However, the roles of CPL in *B. xylophilus* remain unknown.

In this study, the full-length cDNA of three *Bx-cpl*s were cloned, and their amino acid sequences were deduced. Homology analysis showed that *Bx-cpl-1* deduced protein Bx-CPL-1 had a close phylogenetic relationship with the CPL of plant parasitic nematodes, such as *D. destructor*, *M. incognita,* and *H. glycines.* Wang et al. [35] analyzed the homology between the deduced protein of a *cpl* sequence from *D. destructor* (ACT35690), and the CPL of *B. xylophilus* (ACH56225.1) and found that the identities were highly similar. The CPL of *M. incognita* also had a high homology with Bx-CPL-1, which played a crucial role in plant-nematode interaction [30]. The *Bx-cpl-2* and *Bx-cpl-3* deduced proteins (Bx-CPL-2 and Bx-CPL-3) had a close phylogenetic relationship with a CPL of *B. mucronatus* (AID50178.1), which may be related to infection of *B. mucronatus* [36]*.*

ISH enables the investigation of gene expression patterns and gene functions in nematodes [28, 37, 38]. Hashmi et al. [37] reported that the CPL is widely expressed in the head region, intestines, hypodermal cells, and eggshells of *Caenorhabditis elegans*. However, in plant parasitic nematode *M. incognita*, *Mi-cpl-1* was only expressed in the intestinal cells of *M. incognita* [27, 28]. In this study, the localizations of *Bx-cpl-1*, *Bx-cpl-2*, *Bx-cpl-3* were all in the intestine, egg of the female PWN, and the intestine and seminal vesicle of the male PWN. This suggested that the expressions of *Bx-cpl-1*, *Bx-cpl-2*, *Bx-cpl-3* were similar. The Bx-CPL protein might be involved in the digestive process and reproduction of B. xylophilus.

The CPL could regulate the nematode’s development [39]. In this study, three *Bx-cpls* were differently expressed in different developmental stages of *B. xylophilus* and the expressions in eggs were relatively higher than those in juveniles and adults. Hashmi et al. [37] demonstrated that the CPL was essential for embryogenesis and development of *C. elegans*. This suggests that the *Bx-cpls* might play a role in the development of *B. xylophilus,* especially in embryogenesis*.*

The relative expression levels of *Bx-cpl-1,* *Bx-cpl-2*, and *Bx-cpl-3* at PWD development stages were also investigated. The transcript levels of the three *Bx-cpls* from *P. massoniana* (except the transcript level of *Bx-cpl-1* at the last stage) were higher than in the nematodes cultured on *B. cinerea.* This characteristic was similar to many pathogenesis-related genes of *B. xylophilus*, such as pectate lyase genes, cytochrome P450s, UGTs, and ABC transporter genes, which were expressed higher when *B. xylophilus* infected *P. thunbergii*, compared with those cultured on *B. cinerea* [40]. Kang et al. [41] constructed subtractive EST libraries that were specific to the dispersal 4th larval stage (D4S) and the pine-grown propagative mixed stage (PGPS) and found cysteine protease were highly specific to PGPS compared to D4S. The relative expression levels of *Bx-cpl-1,* *Bx-cpl-2*, and *Bx-cpl-3* were highest at the first stage but declined with the development of the PWD. This suggested that *Bx-cpls* might be associated with the parasitic biology of *B. xylophilus* during its propagation within the host pine tree, especially at the first stage of PWD.

RNAi is a means by which double-stranded RNA (dsRNA) induces sequence-specific posttranscriptional gene silencing [42]. It is a very powerful tool for examining the functions of genes in plant nematodes and other organisms [28, 32, 43, 44]. RNAi-induced gene silencing has been achieved in *B. xylophilus* in vitro [21, 38, 45-47]. In this study, the expression levels of *Bx-cpls* treated with *Bx-cpl* dsRNA significantly decreased compared to the control groups, indicating that *Bx-cpl* genes could be silenced. In many parasitic nematodes, CPLs have potential roles in invasion and feeding on host tissues, molting, development, and parasitism [27, 28, 30, 37, 48, 49]. Our results showed that the feeding of PWN weakened; reproduction was reduced; and pathogenicity was lower after silencing *Bx-cpl-1,* *Bx-cpl-2*, or *Bx-cpl-3*, respectively. This suggests that *Bx-cpls* could regulate the nematodes’ reproduction and pathogenicity. Overall, this study focused on the molecular characterization and functional analysis of three *Bx-cpls*. These findings could provide useful information for a better understanding of the molecular mechanism of reproduction and pathogenicity in *B. xylophilus*.

4. Materials and Methods

4.1. Nematode culture and collection

*Bursaphelenchus* *xylophilus* AMA3 isolated from infected *P. thunbergii* in Maanshan, Anhui, China was provided by the Jiangsu Key Laboratory for Prevention and Management of Invasive Species, Nanjing Forestry University.

The PWNs at different developmental stages were collected according to the method described by Shinya et al. [50] with some modifications. The nematodes were cultured on potato dextrose agar (PDA) covered with *Botrytis cinerea* at 25ºC for 4-5 days and isolated with Baermann funnels. The nematodes were washed three times with distilled water and collected by centrifugation at 3500 rpm for 3 min. Approximate 5000 nematodes were placed in a sterilized plate (3 cm diam.) to lay eggs for 4-6 h at 25°C under aseptic conditions. The eggs were collected after the nematodes were discarded by sterile water washing. The juveniles, including the second-juveniles (J2), the third-juveniles (J3) and the forth-juveniles (J4), were obtained after the eggs were cultured on PDA plate containing *B. cinerea* for 30-48h. The adults, including male and female nematodes, were obtained after the eggs were cultured for 84h. The nematodes at different developmental stages were identified under a stereo microscope (Leica MZ95, Germany). Then the nematodes in the same developmental stage were washed three times with distilled water and collected by centrifugation at 3500 rpm for 3 min. The collected nematodes were immediately frozen in liquid nitrogen and stored at −80ºC in a 1.5 mL centrifuge tube for subsequent RNA extraction.

The PWNs at different PWD development stages were collected according to the method described by Ding et al. [51]. The seedlings of *P. massoniana* (2 years old) were disinfected with 75% ethyl alcohol by spraying. Afterwards, 0.5 mL suspensions (about 10,000 mixed-stage nematodes) were pipetted into cutting wounds in *P. massoniana*. Sterile water was used as the control. Then the wounds on *P. massoniana* were sealed by Parafilm. PWNs were collected from three stages based on the corresponding PWD symptoms and inoculation times. The first stage (F): the tips of the pine needles begin to turn brown after pine trees were infected with PWNs. Next, the middle stage (M): half of the needles on pine trees turn brown. The last stage (L): pine needles were completely brown. PWNs cultured on *B. cinerea* served as control. The nematodes were extracted with Baermann funnels and washed three times with distilled water. Then they were, respectively, collected by centrifugation at 3500 rpm for 3 min, immediately frozen in liquid nitrogen and stored at −80ºC in a 1.5 mL centrifuge tube for subsequent RNA extraction.

4.2. RNA extraction, PCR amplification of Bx-cpls and phylogenetic analysis

Total RNA was extracted from the nematodes at each developmental stage and mixed stages using Trizol reagent (Invitrogen, Waltham, MA, USA), measured by ultraviolet absorbance at A260/280 (Eppendorf AG 22331, Hamburg, Germany) and examined by electrophoresis on a 1% agarose gel. The cDNA was synthesized using the TransScript II One-Step gDNA Removal and cDNA Synthesis SuperMix according to the manufacturer’s instructions (TransGen Biotech, Beijing, China). The full-length cDNA sequences of *Bx-cpl-1*, *Bx-cpl-2* and *Bx-cpl-3* were amplified using the 3’-Full RACE Core Set with the PrimeScript™ RTase kit (TaKaRa Biotechnology, Dalian, China) and 5’-Full RACE Kit with TAP (TaKaRa Biotechnology, Dalian, China). Gene-specific primers were used as below: *Bx-cpl-1*: GSP1-1 (3’-Full RACE first round of PCR), GSP1-2 (5’-Full RACE first round of PCR), and GSP1-3 (5’-Full RACE second round of PCR). Gene-specific primers of *Bx-cpl-2*: GSP2-1 (3’-Full RACE first round of PCR) and GSP2-2 (5’-Full RACE first round of PCR). Gene-specific primers of *Bx-cpl-3*: GSP3-1 (3’-Full RACE first round of PCR), GSP3-2 (3’-Full RACE second round of PCR), GSP3-3 (5’-Full RACE first round of PCR), and GSP3-4 (5’-Full RACE second round of PCR) (Table 1). They were designed for 3’ and 5’ RACE amplification based on three partially known sequences of *Bx-cpl-1*，*Bx-cpl-2* and *Bx-cpl-3*, which were obtained from the RNA sequencing results [52]. The PCR product was purified, ligated into the vector *pEASY-T1* (TransGen Biotech, Beijing, China) and transformed into *Escherichia coli Trans1-T1* (*E. coli*) competent cells (TransGen Biotech). The *E. coli* was then incubated overnight at 37ºC on LB plates containing ampicillin. The positive transformants were analyzed by PCR using primers M13F (-47) and M13R (-48) (Table 1). Once the correct clone was identified, the fresh bacterial suspension was submitted to the Nanjing Genscript sequencing company (Nanjing, China) for sequence analysis. The full-length cDNA sequences of *Bx-cpl-1*, *Bx-cpl-2* and *Bx-cpl-3* from *B. xylophilus* were submitted to GenBank and assigned accession numbers as under the accession numbers MG923677, MG923678 and MG923679. A reference and comparison to the genome data available on WormBase Parasite (BioProject PRJEA64437) were performed using blastn and DNAMAN software. Amino acid sequences of homologous Bx-CPL-1，Bx-CPL-2 and Bx-CPL-3 proteins from other species were obtained from NCBI using blastp. Multiple sequence alignment of deduced protein sequences was carried out with ClustalW in MEGA 7 [53]. Phylogenetic relationships among the CPLs were inferred by Maximum Likelihood (ML) method with WAG+G model.

**Table 1.** Polymerase chain reaction (PCR) primers.

| **Name of Primers** | **Sequence (5’ - 3’)** |
| --- | --- |
| **cDNA Cloning of three cathepsin L-like cysteine proteinase genes** | |
| 3’ RACE outer primer | TACCGTCGTTCCACTAGTGATTT |
| 3’ RACE inner primer | CGCGGATCCTCCACTAGTGATTTCACTATAGG |
| GSP1-1 | GCAATGGTGGACTTATGGAC |
| GSP2-1 | AATCCAAGAGCCCCGTTATC |
| GSP3-1 | GCACCTACCGAAGCCGATACTA |
| GSP3-2 | CCACTCCAAGACTACCAAGG |
| 5’RACE outer primer | CATGGCTACATGCTGACAGCCTA |
| 5’RACE inner primer | CGCGGATCCACAGCCTACTGATGATCAGTCGATG |
| GSP1-2 | CTTGACGATCCAGTAGTCGC |
| GSP1-3 | CTCGCCATTTGGTCGCATTT |
| GSP2-2 | GGTTCTATCGCCGACATTCT |
| GSP3-3 | AACCAAAGTGTAGCCCCAAT |
| GSP3-4 | TGACCAAAGCGTTGCGAAGT |
| M13F(−47) | CGCCAGGGTTTTCCCAGTCACGAC |
| M13R(−48) | AGCGGATAACAATTTCACACAGGA |
| **Preparation of template DNA for ISH** | |
| I- *Bx-cpl-1*-F | CCTTTCGCTGAATACCGTCGTCTTA |
| I- *Bx-cpl-1*-R | TGATGACTCAAGCCAGCGGATAACT |
| I- *Bx-cpl-1*-T7-F | TAATACGACTCACTATAGGGCCTTTCGCTGAATACCGTCGTCTTA |
| I- *Bx-cpl-1*-T7-R | TAATACGACTCACTATAGGGTGATGACTCAAGCCAGCGGATAACT |
| I- *Bx-cpl-2*-F | GCTGTGGATGTTGCTACGCTTTTGC |
| I- *Bx-cpl-2*-R | GCTTCTCCGTAGTCCTCTCCCCATT |
| I- *Bx-cpl-2*-T7-F | TAATACGACTCACTATAGGGGCTGTGGATGTTGCTACGCTTTTGC |
| I- *Bx-cpl-2*-T7-R | TAATACGACTCACTATAGGGGCTTCTCCGTAGTCCTCTCCCCATT |
| I- *Bx-cpl-3*-F | ACAGCAGTGCCAAGCCCGCTCAAAT |
| I- *Bx-cpl-3*-R | GTGCTCGGGCATTGATGATTCCTCC |
| I- *Bx-cpl-3*-T7-F | TAATACGACTCACTATAGGGACAGCAGTGCCAAGCCCGCTCAAAT |
| I- *Bx-cpl-3*-T7-R | TAATACGACTCACTATAGGGGTGCTCGGGCATTGATGATTCCTCC |
| **Preparation of template DNA for dsRNA** | |
| *Bx-cpl-1-*T7*-*F | GCCAGTCGTCATCACAAA |
| *Bx-cpl-1-*R | TGTTCCTCATCGGCTTCT |
| *Bx-cpl-1-*F | TAATACGACTCACTATAGGGGCCAGTCGTCATCACAAA |
| *Bx-cpl-1-*T7*-*R | TAATACGACTCACTATAGGGTGTTCCTCATCGGCTTCT |
| *Bx-cpl-2-*T7*-*F | TAATACGACTCACTATAGGGACTAGATCCCAGCGCCACT |
| *Bx-cpl-2-*R | AGCCAACAGTCACGACAGC |
| *Bx-cpl-2-*F | ACTAGATCCCAGCGCCACT |
| *Bx-cpl-2-*T7*-*R | TAATACGACTCACTATAGGGAGCCAACAGTCACGACAGC |
| *Bx-cpl-3-*T7*-*F | TAATACGACTCACTATAGGGAGAGCTTCACAGCAGTGCCAAG |
| *Bx-cpl-3-*R | GTTGAACCTGGTAACTATAGTC |
| *Bx-cpl-3-*F | GCTTCACAGCAGTGCCAAG |
| *Bx-cpl-3-*T7*-*R | TAATACGACTCACTATAGGGAGAGTTGAACCTGGTAACTATAGTC |
| *gfp-*T7*-*F | TAATACGACTCACTATAGGGAGACCATGGCCAACACTTGT |
| *gfp-*R | AGATAATCCCAGCAGCAGTT |
| *gfp-*F | AGACCATGGCCAACACTTGT |
| *gfp-*T7*-*R | TAATACGACTCACTATAGGGAGATAATCCCAGCAGCAGTT |
| **Real time PCR** | |
| q-*Bx-cpl-1-*F | CCAGAAGCCGATGAGGAACA |
| q-*Bx-cpl-1-*R | CCAGTTTTGTAGAGTTGGAAGC |
| q-*Bx-cpl-2-*F | AGTCATCGCTGTAATCTGC |
| q-*Bx-cpl-2-*R | TTGTTGGTGCCATAAGTG |
| q-*Bx-cpl-3-*F | CTATAACGGAGTCACCTCCAT |
| q-*Bx-cpl-3-*R | TGCTCTTCACTGAGATCCAGT |
| Actin-F | GCAACACGGAGTTCGTTGTAGA |
| Actin-R | GTATCGTCACCAACTGGGATGA |

4.3. In situ hybridization (ISH)

The ISH probe templates were generated by PCR based on the full-length cDNA sequences of *Bx-cpl-1*, *Bx-cpl-2* and *Bx-cpl-3* with the specific primer pairs (Table 1). The DIG-labeled sense RNA probes and antisense RNA probes were synthesized from the PCR products of *Bx-cpls* using the DIG Northern Starter Kit (Roche Diagnostics, Mannheim, Germany)[54]. The nematodes were treated, and hybridizations were performed as described by De Boer et al.[55] using DIG High Prime DNA Labeling and Detection Starter Kit I (Roche Diagnostics, Mannheim, Germany). The control group were used the DIG-labeled sense RNA probes. Finally, the nematodes were examined and photographed using a Zeiss Axio Image M2 microscope (Zeiss MicroImaging GmbH, Oberkochen, Germany).

4.4. Synthesis of Bx-cpl dsRNA and interference

Double-stranded RNA (dsRNA) was synthesized using the MEGscript RNAi Kit (Ambion Inc., Austin, TX, USA) with the specific primers containing the T7 promoter (Table 1). The non-endogenous control dsRNA (the green ﬂuorescent protein gene, *gfp*) was synthesized using the specific primers *gfp-*T7*-*F/*gfp-*R and *gfp-*F/*gfp-*T7*-*R (Table 1). The RNAi soaking method was performed according to Urwin *et al.* [32]. Approximate 3,000 individuals (a mixture of juveniles and adults) of freshly cultured nematodes were soaked in dsRNA solution (800 ng/µL) after being washed with distilled water for 3 times at 3500 rpm for 3 min, and then incubated at 180 rpm for 48 h at 20ºC. The nematodes soaked in the corresponding *gfp* dsRNA and ddH_2_O were controls. Each treatment had three replicates. Samples from each treatment were washed thoroughly with ddH_2_O several times after soaking and then used for additional experiments.

4.5. The qPCR and expression analysis of Bx-cpls

Expressions of *Bx-cpl*s were analyzed using real-time quantitative PCR (qPCR). Total RNA were extracted from 3000 nematodes at each developmental stage and mixed stages using Trizol reagent (Invitrogen, Waltham, MA, USA). The RNA quantity and integrity were checked as previously described. The cDNA was synthesized using TransScript II One-Step gDNA Removal and cDNA Synthesis SuperMix following the manufacturer’s protocol (TransGen Biotech, Beijing, China). Specific primers were designed from the cDNA sequence of target genes using the Primer Premier 5.0 (Table 1). Actin gene was amplified as a reference gene using the primers Actin-F/Actin-R (Table 1). The qPCR was performed on ABI Prism 7500 (Applied Biosystems, Foster City, CA, USA) using SYBR Green Master Mix (Vazyme, Nanjing, China). The initial data analysis was performed using ABI Prism 7500 software and the 2^−ΔΔCt^ method. All experiments were performed in triplicate with three biological replicates.

4.6. Analysis of reproduction and pathogenicity of B. xylophilus after RNAi

About 200 nematodes treated with *Bx-cpl* dsRNA were cultured on a PDA plate with *B. cinerea* at 25°C for 6 days. The ddH_2_O and *gfp* dsRNA were used as controls. Each treatment had three replicates. The feeding of *B. xylophilus* was observed and photographed periodically. Subsequently, the nematodes were washed off the plates using the Baermann funnel. The reproduction of nematodes was counted with an optical stereo microscope (Leica MZ95, Germany). In order to determine the pathogenicity of *B. xylophilus* after RNAi, nearly 2000 nematodes soaked in *Bx-cpl* dsRNA, *gfp* dsRNA or ddH_2_O without dsRNA were inoculated into each 4-year-old *P. massoniana* seedling. ddH_2_O without nematodes was used as inoculation control. Each treatment contained four replicates. The inoculated seedlings were placed in the greenhouse. Photographs were taken regularly to record infection state of the seedlings. PWD symptoms were evaluated and categorized as 0–4 [56]. The categories were as follows: 0 = all needles were green; 1 = 0%–25% of needles were discolored and turned yellow; 2 = 25%–50% of needles turned yellow; 3 = 50%–75% of needles turned yellow; and 4 = 75%–100% of needles turned yellow. The infection rates and the disease severity index (DSI) were calculated with the formula as follow:

$$\text{Infection rates=}\frac{\sum\text{Number of infected plants}}{\text{Total number of plants}}\text{×100\%}$$

$$DSI=\frac{\sum Number of disease plants\times symptom stage}{Total number of plant\times highest symtom stage}\times100$$

4.7. Statistical analysis

All data were presented as the means ±standard deviation (Mean ± S.D.). All parameters were calculated using Microsoft Excel. The statistical significance was determined using SPSS Statistics 17.0 software (IBM China Company Ltd., Beijing, China) with one-way analysis of variance (ANOVA) and (variance analysis) T-test. The level of significance was P < 0.05.

**Supplementary Materials:** Figure S1. Full length cDNA sequences and deduced amino acid sequences of *Bx-cpls.* *Bx-cpl-1* (**A**); *Bx-cpl-2* (**B**); *Bx-cpl-3*(**C**). Figure S2: Comparison of *Bx-cpls* gene sequences to genome data of *B. xylophilus*. *Bx-cpl-1* gene (**A**); *Bx-cpl-2* gene (**B**); *Bx-cpl-3* gene (**C**). The intron sequences were underlined.

**Author Contributions:** conceptualization, Q.X. and X.W.; methodology, Q.X. and X.W.; validation, Q.X., X.W. and L.D..; formal analysis, Q.X.; investigation, Q.X., W.Z. and M.W.; resources, X.W.; data curation, Q.X., W.Z. and M.W.; writing—original draft preparation, Q.X.; writing—review and editing, Q.X. and X.W.; visualization, Q.X..; supervision, X.W.; project administration, Q.X. and X.W.; funding acquisition, X.W.

**Funding:** This research was funded by the Jiangsu Provincial Agricultural Science and Technology Innovation Fund (CX (16) 1005), the Priority Academic Program Development of Jiangsu Higher Education Institutions (PAPD) and Innovation Plan for Graduate Students of Jiangsu, China (KYZZ16_0315).

**Acknowledgments:** We are grateful to Dr. De-Wei Li, The Connecticut Agricultural Experiment Station, USA for reviewing the manuscript.

**Conflicts of Interest:** The authors declare no conflict of interest. The funders had no role in the design of the study; in the collection, analyses, or interpretation of data; in the writing of the manuscript, or in the decision to publish the results.

References

1. Shinya, R.; Morisaka, H.; Takeuchi, Y.; Futai, K.; Ueda, M. Making headway in understanding pine wilt disease: what do we perceive in the postgenomic era? *J Biosci Bioeng*. **2013**, *116*, 1-8, doi: 10.1016/j.jbiosc.2013.01.003.
2. Dwinell, L.D. First report of pinewood nematode (*Bursaphelenchus xylophilus*) in Mexico. *Plant Dis*. **1993**, *77*, 846A, doi: 10.1094/PD-77-0846A.
3. Mamiya, Y. History of pine wilt disease in Japan. *J Nematol*. **1988**, *20*, 219-226, pmcid: PMC2618808.
4. Zhang, K.; Liang, J.; Yan, D.H.; Zhang, X.Y. Research advances of pine wood nematode disease in China. *World Forestry Res*. **2010**, *23*, 59-63, doi: 10.13348/j.cnki.sjlyyj.2010.03.008.
5. Yi, C.K.; Byun, B.H.; Park, J.D.; Yang, S.I.; Chang, K.H. First finding of the pine wood nematode, *Bursaphelenchus xylophilus* (Steiner et Buhrer) Nickle and its insect vector in Korea. *Res Rep For Res In*. **1989**,141-149.
6. Mota, M.M.; Braasch, H.; Bravo, M.A.; Penas, A.C.; Burgermeister, W.; Metge, K.; Sousa, E. First report of *Bursaphelenchus xylophilus* in Portugal and in Europe. *Nematol*. **1999**, *1*, 727-734, doi: 10.1163/156854199508757.
7. Abelleira, A.; Picoaga, A.; Mansilla, J.P.; Aguin, O. Detection of *Bursaphelenchus xylophilus*, causal agent of pine wilt disease on *Pinus pinaster* in northwestern Spain. *Plant Dis*. **2011**, *95*, 776-776, doi: 10.1094/PDIS-12-10-0902.
8. Khan, F.A.; Gbadegesin, R.A. On the occurrence of nematode induced pine wilt disease in Nigeria. *Pak J Nematol*. **1991**, *57*, 162-164.
9. Kikuchi, T.; Aikawa, T.; Kosaka, H.; Pritchard, L.; Ogura, N.; Jones, J.T. Expressed sequence tag (EST) analysis of the pine wood nematode *Bursaphelenchus xylophilus* and *B. mucronatus*. *Mol Biochem Parasitol*. **2007**, *155*, 9-17, doi: 10.1016/j.molbiopara.2007.05.002.
10. Kikuchi, T.; Cotton, J.A.; Dalzell, J.J.; Hasegawa, K.; Kanzaki, N.; McVeigh, P.; Takanashi, T.; Tsai, I.J.; Assefa, S.A.; Cock, P.J.; et al. Genomic insights into the origin of parasitism in the emerging plant pathogen *Bursaphelenchus xylophilus*. *Plos Pathog*. **2011**, *7*, e1002219, doi: 10.1371/journal.ppat.1002219.
11. Shinya, R.; Morisaka, H.; Kikuchi, T.; Takeuchi, Y.; Ueda, M.; Futai, K. Secretome Analysis of the pine wood nematode *Bursaphelenchus xylophilus* reveals the tangled roots of parasitism and its potential for molecular mimicry. *PLoS One*. **2013**, *8*, e67377, doi: 10.1371/journal.pone.0067377.
12. Tsai, I.J.; Tanaka, R.; Kanzaki, N.; Akiba, M.; Yokoi, T.; Espada, M.; Jones, J.T.; Kikuchi, T. Transcriptional and morphological changes in the transition from mycetophagous to phytophagous phase in the plant-parasitic nematode *Bursaphelenchus xylophilus*. *Mol Plant Pathol*. **2016**, *17*, 77-83, doi: 10.1111/mpp.12261.
13. Cardoso, J.; Anjo, S.; Fonseca, L.; Egas, C.; Manadas, B.; Abrantes, I. *Bursaphelenchus xylophilus* and *B. mucronatus* secretomes: a comparative proteomic analysis. *Sci Rep*. **2016**, *6*, 39007, doi: 10.1038/srep39007.
14. Kikuchi, T.; Jones, J.T.; Aikawa, T.; Kosaka, H.; Ogura, N. A family of glycosyl hydrolase family 45 cellulases from the pine wood nematode *Bursaphelenchus xylophilus*. *FEBS Lett*. **2004**, *572*, 201-205, doi: 10.1016/j.febslet.2004.07.039.
15. Zhang, L.; Fan, Y.; Zheng, H.; Du, F.; Zhang, K.Q.; Huang, X.; Wang, L.2.; Zhang, M.; Niu, Q. Isolation and characterization of a novel endoglucanase from a *Bursaphelenchus xylophilus* metagenomic library. *PLoS One*. **2013**, *8*, e82437, doi: 10.1371/journal.pone.0082437.
16. Kikuchi, T.; Shibuya, H.; Aikawa, T.; Jones, J.T. Cloning and characterization of pectate lyases expressed in the esophageal gland of the pine wood nematode *Bursaphelenchus xylophilus*. *Mol Plant Microbe Interact*. **2006**, *19*, 280-287, doi: 10.1094/MPMI-19-0280.
17. Lee, D.W.; Kang, J.S.; Jung, C.S.; Han, H.R.; Moon, Y.S.; Park, S.J.; Lee, S.H.; Koh, Y.H. Identification and biochemical analysis of a novel pectate lyase 3 gene in *Bursaphelenchus xylophilus*. *J Asia-Pac Entomol*. **2013**, *16*, 335-342, doi: 10.1016/j.aspen.2013.04.016.
18. Kikuchi, T.; Li, H.M.; Karim, N.; Kennedy, M.W.; Moens, M.; Jones, J.T. Identification of putative expansin-like genes from the pine wood nematode, *Bursaphelenchus xylophilus*, and evolution of the expansin gene family within the nematoda. *Nematol*. **2009**, *11*, 355-364, doi: 10.1163/156854109X446953.
19. Kim, Y.H.; Kim, A.Y.; Choi, B.H.; Han, H.R.; Koh, Y.H. ExpansinB3 as a marker for detecting pine wood nematode-infected pine trees. *J Asia-Pac Entomo*l. **2017**, *20*, 1228-1233, doi: 10.1016/j.aspen.2017.08.029.
20. Lin, S.F.; Jian, H.; Zhao, H.J.; Yang, D.; Liu, Q. Cloning and characterization of a venom allergen-like protein gene cluster from the pinewood nematode *Bursaphelenchus xylophilus*. *Exp Parasitol*. **2011**, *127*, 440-447, doi: 10.1016/j.exppara.2010.10.013.
21. Xu, X.L.; Wu, X.Q.; Ye, J.R.; Huang, L. Molecular characterization and functional analysis of three pathogenesis-related cytochrome P450 genes from *Bursaphelenchus xylophilus* (Tylenchida, Aphelenchoidoidea). *Int J Mol Sci*. **2015**, *16*, 5216-5234, doi: 10.3390/ijms16035216.
22. Rhoads, M.L.; Fetterer, R.H. Extracellular matrix: A tool for defining the extracorporeal function of parasite proteases. *Parasitol Today*. **1997**, *13*, 119-122, doi: 10.1016/S0169-4758(96)40011-4.
23. Malagón, D.; Benítez, R.; Kašný, M.; Adroher, F.J. Peptidases in parasitic nematodes. A review. In *Parasites: Ecology, Diseases and Management*, Erzinger GS, Ed.; Nova Science Publishers Inc: New York, USA, 2013; pp. 61-102; 978-1-62257-692-0.
24. Sajid, M.; McKerrow, J.H. Cysteine proteases of parasitic organisms. *Mol Biochem Parasitol*. **2002**, *120*, 1-21, doi: 10.1016/S0166-6851(01)00438-8.
25. Britton, C.; Murray, L. A cathepsin L protease essential for *Caenorhabditis elegans* embryogenesis is functionally conserved in parasitic nematodes. *Mol Biochem Parasitol*. **2002**, *122*, 21-33, doi: 10.1016/S0166-6851(02)00066-X.
26. Urwin, P.E.; Lilley, C.J.; McPherson, M.J.; Atkinson, H.J. Characterization of two cDNAs encoding cysteine proteinases from the soybean cyst nematode *Heterodera glycines*. *Parasitol*. **1997**, *114*, 605-613, doi: 10.1016/S0166-6851(97)00116-3
27. Neveu, C.; Abad, P.; Castagnone-Sereno, P. Molecular cloning and characterization of an intestinal cathepsin L protease from the plant-parasitic nematode *Meloidogyne incognita*. *Physiol Mol Plant Pathol*. **2003**, *63*, 159-165, doi: 10.1016/j.pmpp.2003.10.005.
28. Shingles, J.; Lilley, C.J.; Atkinson, H.J.; Urwin, P.E. *Meloidogyne incognita*: molecular and biochemical characterisation of a cathepsin L cysteine proteinase and the effect on parasitism following RNAi. *Exp Parasitol*. **2007**, *115*, 114-120, doi: 10.1016/j.exppara.2006.07.008.
29. Neveu, C.; Jaubert, S.; Abad, P.; Castagnone-Sereno, P. A set of genes differentially expressed between avirulent and virulent *Meloidogyne incognita* near-isogenic lines encode secreted proteins. *Mol Plant Microbe Interact*. **2003**, *16*, 1077, doi: 10.1094/MPMI.2003.16.12.1077.
30. Dutta, T.K.; Papolu, P.K.; Banakar, P.; Choudhary, D.; Sirohi, A.; Rao, U. Tomato transgenic plants expressing hairpin construct of a nematode protease gene conferred enhanced resistance to root-knot nematodes. *Front Microbiol*. **2015**, *6*, 260, doi: 10.3389/fmicb.2015.00260.
31. Duarte, A.; Maleita, C.; Tiago, I.; Curtis, R.; Abrantes, I. Molecular characterization of putative parasitism genes in the plant-parasitic nematode *Meloidogyne hispanica*. *J Helminthol*. **2016**, *90*, 28-38, doi: 10.1017/S0022149X1400073X.
32. Urwin, P.E.; Lilley, C.J.; Atkinson, H.J. Ingestion of double-stranded RNA by preparasitic juvenile cyst nematodes leads to RNA interference. *Mol Plant Microbe Interact*. **2002**, *15*, 747-752, doi: 10.1094/MPMI.2002.15.8.747.
33. Dalton, J.P.; Neill, S.O.; Stack, C.; Collins, P.; Walshe, A.; Sekiya, M.; Doyle, S.; Mulcahy, G.; Hoyle, D.; Khaznadji, E.; et al. *Fasciola hepatica* cathepsin L-like proteases: biology, function, and potential in the development of first generation liver fluke vaccines. *Int J Parasitol*. **2003**, *33*, 1173-1181, doi: 10.1016/S0020-7519(03)00171-1.
34. Corvo, I.; Cancela, M.; Cappetta, M.; Pi-Denis, N.; Tort, J.F.; Roche, L. The major cathepsin L secreted by the invasive juvenile *Fasciola hepatica* prefers proline in the S2 subsite and can cleave collagen. *Mol Biochem Parasit*. **2009**, *167*, 41, doi: 10.1016/j.molbiopara.2009.04.005.
35. Wang, G.F.; Peng, D.L.; Sun, J.H.; Huang, W.K.; Peng, H.; Long, H.B. Cloning and sequence analysis of a new cathepsin L-like cysteine proteinase gene from *Ditylenchus destructor*. *Chinese J Biotechnol*. **2011**, *27*, 60-68, doi: 10.13345/j.cjb.2011.01.010.
36. Pan, Y.Y.; Huang, L.; Wu, X.Q. Bioinformatic and expression analysis of a cathepsin gene Bmcath1 in *Bursaphelechus mucronatus*. *J Nanjing For Univ*. **2015**, *39*, 12-16, doi: 10.3969/j.issn.1000-2006.2015.06.003.
37. Hashmi, S.; Britton, C.; Liu, J.; Guiliano, D.B.; Oksov, Y.; Lustigman, S. Cathepsin L is essential for embryogenesis and development of *Caenorhabditis elegans*. *J Biol Chem*. **2002**, *277*, 3477, doi: 10.1074/jbc.M106117200.
38. Deng, L.N.; Wu, X.Q.; Ye, J.R.; Xue, Q. Identification of autophagy in the pine wood nematode *Bursaphelenchus xylophilus* and the molecular characterization and functional analysis of two novel autophagy-related genes, BxATG1 and BxATG8. *Int J Mol Sci*. **2016**, *17*, 279, doi: 10.3390/ijms17030279.
39. Rhoads, M.L.; Fetterer, R.H. Developmentally regulated secretion of cathepsin L-like cysteine proteases by *Haemonchus contortus*. *J Parasitol*. **1995**, *81*, 505-512, doi: 10.2307/3283844.
40. Qiu, X.W.; Wu, X.Q.; Huang, L.; Tian, M.Q.; Ye, J.R. Specifically expressed genes of the nematode *Bursaphelenchus xylophilus* involved with early interactions with pine trees. *PLoS One*. **2013**, *8*, e78063, doi: 10.1371/journal.pone.0078063.
41. Kang, J.S.; Lee, H.; Moon, I.S.; Lee, Y.; Koh, Y.H.; Je, Y.H.; Lim, K.J.; Lee, S.H. Construction and characterization of subtractive stage-specific expressed sequence tag (EST) libraries of the pinewood nematode *Bursaphelenchus xylophilus*. *Genomics*. **2009**, *94*, 70-77, doi: 10.1016/j.ygeno.2009.03.001.
42. Fire, A.; Xu, S.; Montgomery, M.K.; Kostas, S.A.; Driver, S.E.; Mello, C.C. Potent and specific genetic interference by double-stranded RNA in *Caenorhabditis elegans*. *Nature*. **1998**, *391*, 806-811, doi: 10.1038/35888.
43. Rosso, M.N.; Dubrana, M.P.; Cimbolini, N.; Jaubert, S.; Abad, P. Application of RNA interference to root-knot nematode genes encoding esophageal gland proteins. *Mol Plant Microbe Interact*. **2005**, *18*, 615-620, doi: 10.1094/MPMI-18-0615.
44. Li, Y.; Xie, H.; Xu, C.L.; Li, D.L.; Zhang, C. RNAi effect of cathepsin B gene on reproduction of *Radopholus similis*. *Scientia Agricultura Sinica*. **2010**, *43*, 1608-1616, doi: 10.3864/j.issn.0578-1752.2010.08.009.
45. Li, X.D.; Zhuo, K.; Luo, M.; Sun, L.; Liao, J. Molecular cloning and characterization of a calreticulin cDNA from the pinewood nematode *Bursaphelenchus xylophilus*. *Exp Parasitol*. **2011**, *128*, 121-126, doi: 10.1016/j.exppara.2011.02.017.
46. Wang, X.R.; Cheng, X.; Li, Y.D.; Zhang, J.A.; Zhang, Z.F.; Wu, H.R. Cloning arginine kinase gene and its RNAi in *Bursaphelenchus xylophilus* causing pine wilt disease. *Eur J Plant Pathol*. **2012**, *134*, 521-532, doi: 10.1007/s10658-012-0035-0.
47. Cardoso, J.M.S.; Fonseca, L.; Gomes, P.; Egas, C.; Abrantes, I. Molecular characterization and functional analysis of a calponin gene from the pinewood nematode. *Forest Pathol*. **2015**, *45*, 467-73, doi: 10.1111/efp.12196.
48. Koiwa, H.; Shade, R.E.; Zhu-Salzman, K.; D'Urzo, M.P.; Murdock, L.L.; Bressan, R.A.; Hasegawa, P.M. A plant defensive cystatin (soya cystatin) targets cathepsin L-like digestive cysteine proteinases (DvCALs) in the larval midgut of western corn rootworm (*Diabrotica virgifera*). *FEBS Lett*. **2000**, *471*, 67-70, doi: 10.1016/S0014-5793(00)01368-5.
49. Lustigman, S.; Zhang, J.; Liu, J.; Oksov, Y.; Hashmi, S. RNA interference targeting cathepsin L and Z-like cysteine proteases of *Onchocerca volvulus* confirmed their essential function during L3 molting. *Mol Biochem Parasitol*. **2004**, *138*, 165-170, doi: 10.1016/j.molbiopara.2004.08.003.
50. Shinya, R.; Takeuchi, Y.; Futai, K. A technique for separating the developmental stages of the propagative form of the pine wood nematode, *Bursaphelenchus xylophilus*. *Nematol*. **2009**, *11*, 305-307, doi: 10.1163/156854108X399164.
51. Ding, X.L.; Ye, J.R.; Wu, X.Q.; Huang, L.; Zhu, L.H.; Lin, S.X. Deep sequencing analyses of pine wood nematode *Bursaphelenchus xylophilus* microRNAs reveal distinct miRNA expression patterns during the pathological process of pine wilt disease. *Gene*. **2015**, *555*, 346-356, doi: 10.1016/j.gene.2014.11.030.
52. He, L.X.; Wu, X.Q.; Xue, Q.; Qiu, X.W. Effects of endobacterium (*Stenotrophomonas maltophilia*) on pathogenesis-related gene expression of pine wood nematode (*Bursaphelenchus xylophilus*) and pine wilt disease. *Int J Mol Sci*. **2016**, *17*, 778, doi: 10.3390/ijms17060778.
53. Kumar, S.; Stecher, G.; Tamura, K. MEGA7: Molecular Evolutionary Genetics Analysis version 7.0 for bigger datasets. *Mol Biol and Evol* **2016**, *33*, 1870-1874, doi:10.1093/molbev/msw054.
54. Regina, W.; Corinna, W.; Alexandra, F.; Jarutat, T.; Astrid, H.; Tobias, B.; William, D.; Barbara, R. A method for high quality digoxigenin-labeled rna probes for in situ hybridization. http://www.ebiotrade.com/custom/upload/140506/1.pdf.
55. De Boer, J.M.; Yan, Y.; Smant, G.; Davis, E.L.; Baum, T.J. In-situ hybridization to messenger RNA in *Heterodera glycines*. *J Nematol*. **1998**, *30*, 309-312, pmcid: PMC2620305.
56. Yu, L.Z.; Wu, X.Q.; Ye, J.R.; Zhang, S.N.; Wang, C. NOS-like-mediated nitric oxide is involved in *Pinus thunbergii* response to the invasion of *Bursaphelenchus xylophilus*. *Plant Cell Rep*. 2012, *31*, 1813-1821, doi: 10.1007/s00299-012-1294-0.

© 2018 by the authors. Submitted for possible open access publication under the terms and conditions of the Creative Commons Attribution (CC BY) license (http://creativecommons.org/licenses/by/4.0/).
