# Supplementary figures and images for: Cathepsin L-like Cysteine Proteinase Genes Are Associated with the Development and Pathogenicity of Pine Wood Nematode, Bursaphelenchus xylophilus
Source: Int J Mol Sci. 2019 Jan 8;20(1):215. doi: 10.3390/ijms20010215 (PMC6337200; doi:10.3390/ijms20010215)

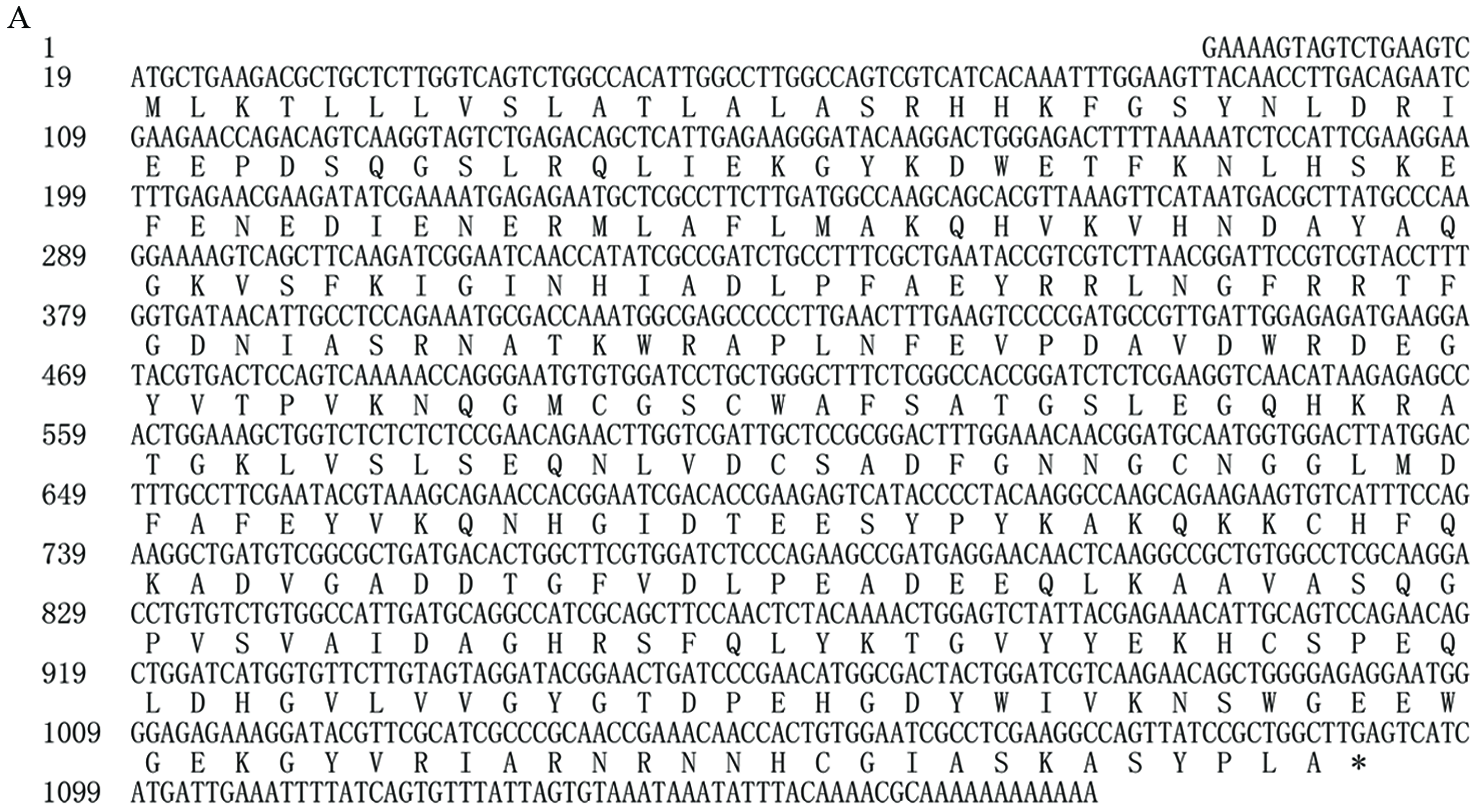

Supplement: Supplementary file 1 [file ijms-20-00215-s001.zip › ijms-418529-supp/Supplementary File/S1A Fig.tif]

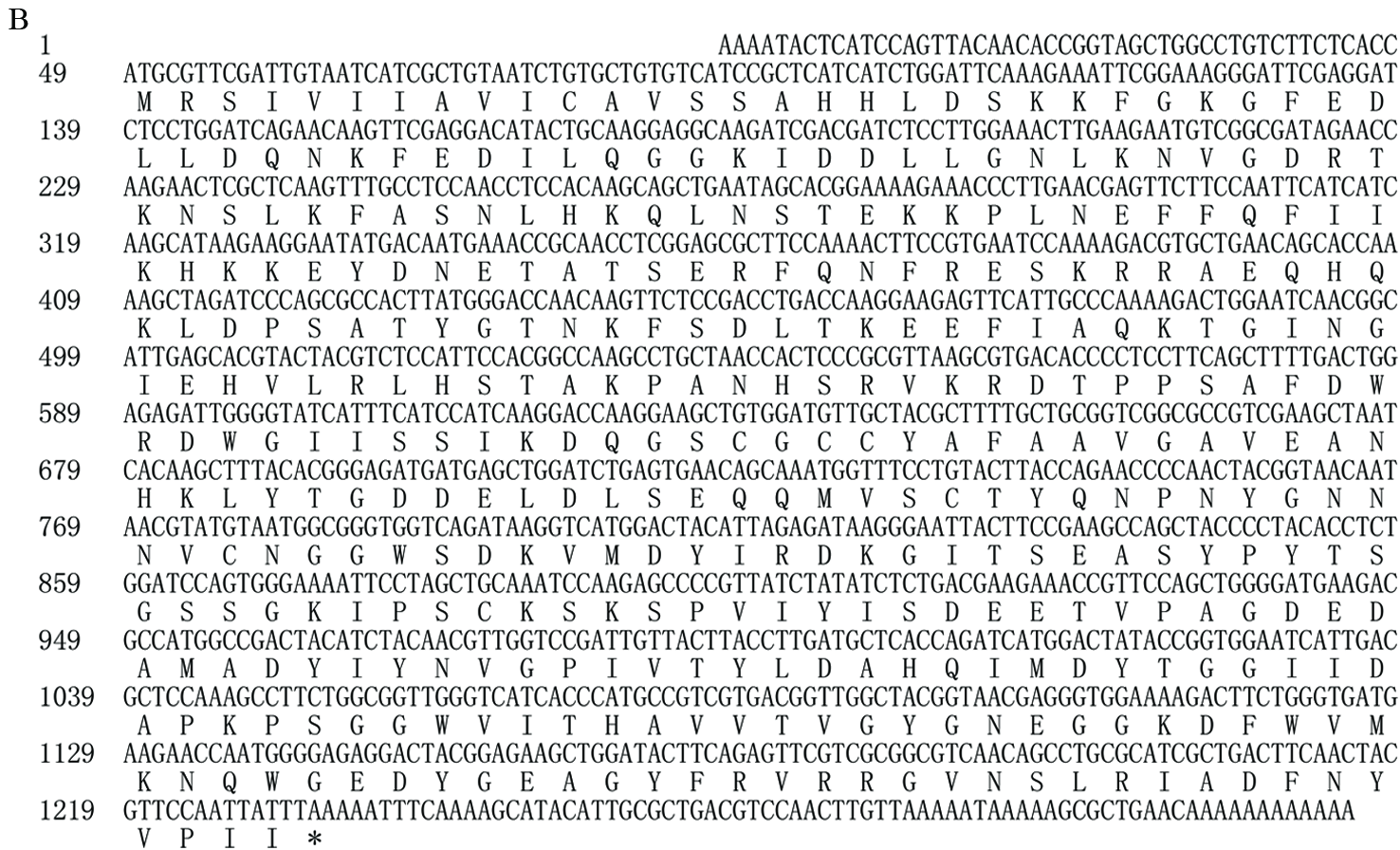

Supplement: Supplementary file 1 [file ijms-20-00215-s001.zip › ijms-418529-supp/Supplementary File/S1B Fig.tif]

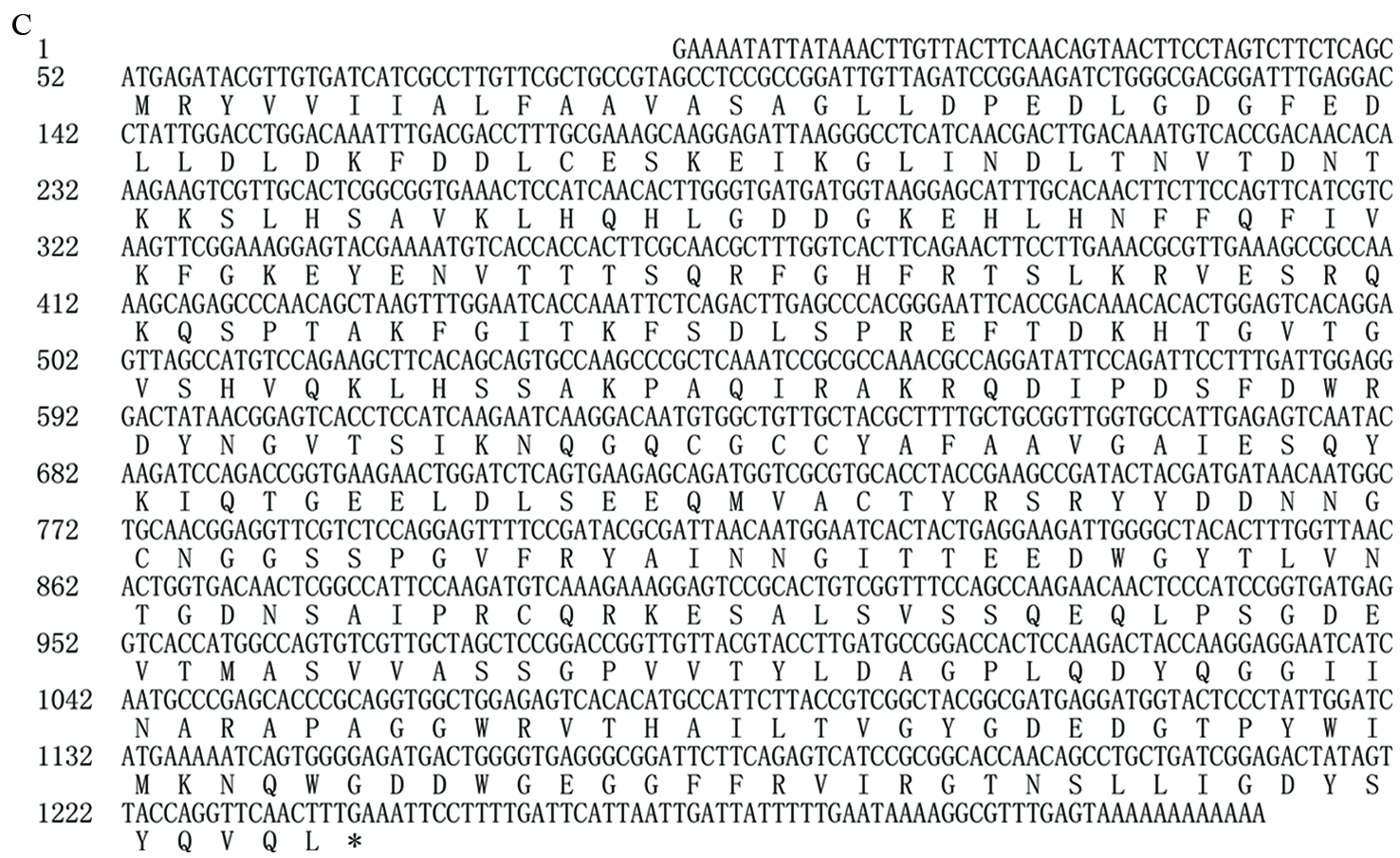

Supplement: Supplementary file 1 [file ijms-20-00215-s001.zip › ijms-418529-supp/Supplementary File/S1C Fig.tif]

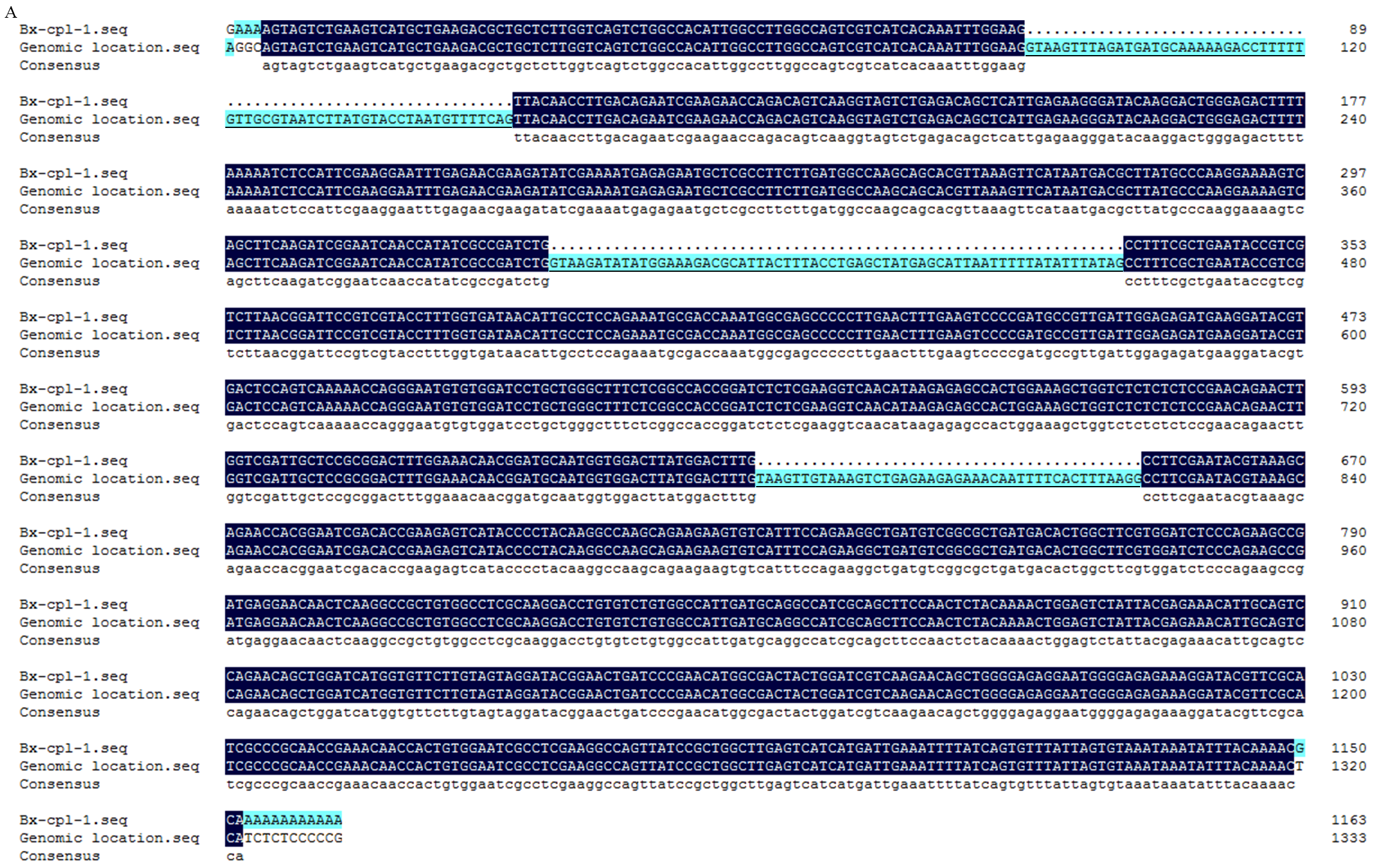

Supplement: Supplementary file 1 [file ijms-20-00215-s001.zip › ijms-418529-supp/Supplementary File/S2A Fig.tif]

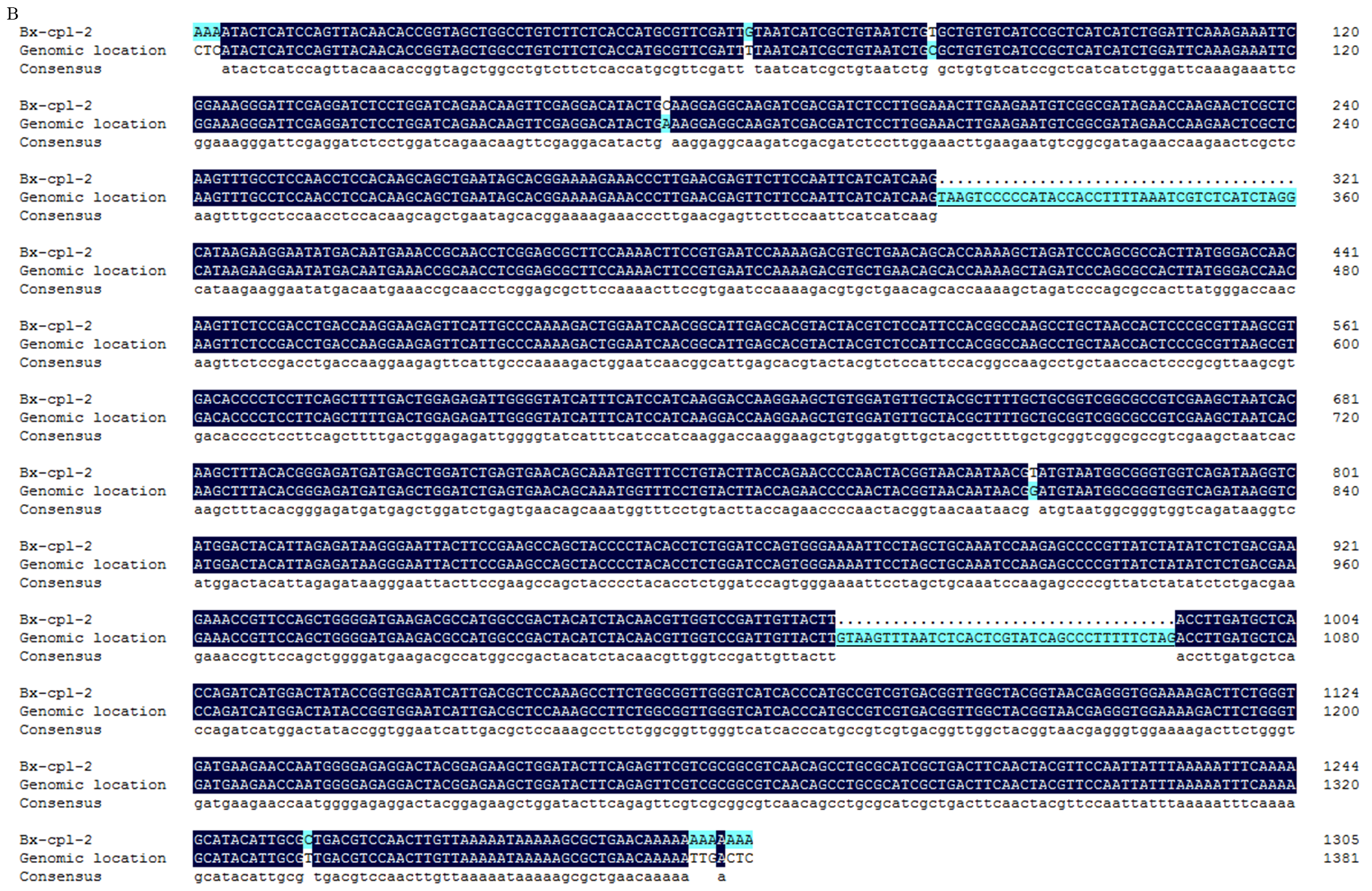

Supplement: Supplementary file 1 [file ijms-20-00215-s001.zip › ijms-418529-supp/Supplementary File/S2B Fig.tif]

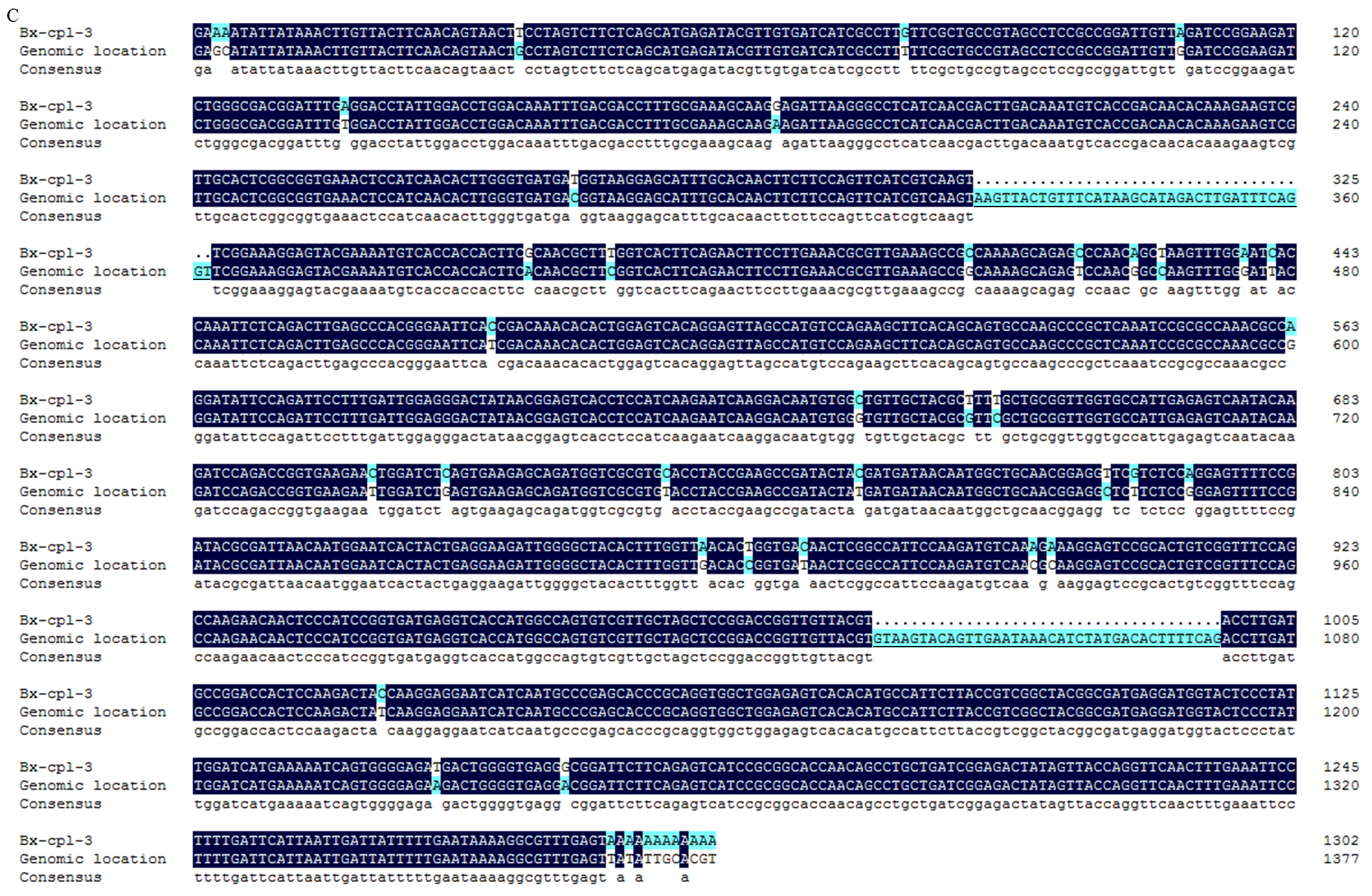

Supplement: Supplementary file 1 [file ijms-20-00215-s001.zip › ijms-418529-supp/Supplementary File/S2C Fig.tif]
