# Supplementary material for: Cathepsin L-like Cysteine Proteinase Genes Are Associated with the Development and Pathogenicity of Pine Wood Nematode, Bursaphelenchus xylophilus
Source: Int J Mol Sci. 2019 Jan 8;20(1):215. doi: 10.3390/ijms20010215 (PMC6337200; doi:10.3390/ijms20010215)
Supplement: Supplementary file 1 [file ijms-20-00215-s001.zip › Supplementary File/respond reviewer 1.docx]

Response to Reviewer 1 Comments

Dear reviewer,
Thank you for your careful comments to improve the manuscript. The manuscript has been revised based on your comments and all corresponding changes made to the manuscript are indicated by using track changes. The point-by-point responses to your comments are listed below.

**Point 1:** Line 22: were 1163 bp, 1305 bp and 1302 bp, respectively.

Line 62: The expressions of *Bx-cpls* in

Line 63: The roles of *Bx-cpls*

**Response 1:** We have revised them in the manuscript.

**Point 2:** Figure 2: please use protein names Bx-CPL-1, Bx-CPL-2 and Bx-CPL-3 to replace the number 1, 2 and 3, respectively in the tree.  Please cite the reference when use the software MEGA7.

**Response 2:** We have used protein names Bx-CPL-1, Bx-CPL-2 and Bx-CPL-3 to replace the number 1, 2 and 3, respectively in the tree in Figure 2. The reference about software MEGA7 has been cited.

**Point 3:** In the results of 2.1, the localization of *Bx-cpl* in *B. xylophilus* is not accurate and faint by ISH. The authors may use GFP or YFP-fused CPL proteins to detect the protein localization by microscope at different development and infection stages.

**Response 3:** Our previous experiments in our laboratory showed that *B. xylophilus* had strong autofluorescence. GFP or YFP-fused CPL proteins might be be confused with autofluorescence. ISH was used to analyze the tissue specificity in many nematodes. Therefore, we used ISH to detect the gene localization by microscope.

**Point 4:** Figure 5: The authors need to present the figures of symptoms in *P. massoniana* after inoculation with nematodes, corresponding to first stage (F), middle stage (M) and last stage (L).

**Response 4:** We have presented the figures of symptoms in *P. massoniana* after inoculation with nematodes, corresponding to first stage (F), middle stage (M) and last stage (L) in our revised manuscript.

**Point 5:** Figure 6 in result 2.5. Under each treatment, the authors need to detect the expression of all three *cpl* genes to confirm whether off-target effect of siRNA exists. For example, whether dsRNA of *Bx-cpl-1* can target *Bx-cpl-2* and *Bx-cpl-3* for degradation?

**Response 5:** We have detected the expression of all three *cpl* genes under each treatment, and added it in our revised manuscript.

**Point 6:** Result 2.6: the authors need to detect the expression levels of all three *cpl* genes under each treatment of five treatments (ddH_2_O and dsRNA (gfp, Bx-cpl-1, Bx-cpl-2 and Bx-cpl-3)). Moreover, did the authors try the silencing of two or three *cpl* genes together?

**Response 6:** We have detected the expression levels of all three *cpl* genes under each treatment of five treatments after cultivation on *B. cinerea* six days, and added it in our revised manuscript. Moreover, we try the silencing of two or three *cpl* genes together. However, perhaps because of a faulty method, the target gene was not silenced. So we didn’t present the result.

**Point 7:** Figure 8: the authors need to present the silencing efficiency of each *cpl* genes in this experiment to confirm that the different disease symptoms indeed were resulted from the knockdown of *cpl* genes.

**Response 7:** The result 2.5 was silencing efficiency of each *cpl* genes in this experiment. Due to the timeliness of rani, we speculated that the silencing efficiency would gradually decline after inoculation. Even so, it could be explained that the different disease symptoms were resulted from the knockdown of *cpl* genes. Therefore, we didn’t detect the silencing efficiency of each *cpl* genes after inoculation.
