# Supplementary material for: Cathepsin L-like Cysteine Proteinase Genes Are Associated with the Development and Pathogenicity of Pine Wood Nematode, Bursaphelenchus xylophilus
Source: Int J Mol Sci. 2019 Jan 8;20(1):215. doi: 10.3390/ijms20010215 (PMC6337200; doi:10.3390/ijms20010215)
Supplement: Supplementary file 1 [file ijms-20-00215-s001.zip › Supplementary File/respond reviewer 2.docx]

Response to Reviewer 2 Comments

Dear reviewer,
Thank you for your careful comments to improve the manuscript. The manuscript has been revised based on your comments and all corresponding changes made to the manuscript are indicated by using track changes. The point-by-point responses to your comments are listed below.

**Point 1:** Fig. 1 could be replaced by a figure which shows bands after gel eletrophoresis

**Response 1:** We have replaced Fig. 1 by a figure which shows bands after gel eletrophoresis.

**Point 2:** ddH_2_O: here 2 must be lowercase. L141: have to be ddH_2_O; also in Figure 2; L149, L153 etc.

**Response 2:** We have revised it in the manuscript.

**Point 3:** L138-have to write Botrytis cinerea instead of B. cinerea

**Response 3:** We have revised it in the manuscript.

**Point 4:** Gene names sometime written in italic and sometime non italic. Italic would be better. Eg. L156 Bx-cpl dsRNA have to be italic. also in L279

**Response 4:** We have written gene names in italic.

**Point 5:** L164-Pinus massoniana instead of P. massoniana

**Response 5:** We have revised it in the manuscript.

**Point 6:** L276: ...were submitted to GenBank and assigned accession numbers as ....(Cause: accession number given after proper submission)

**Response 6:** We have revised it in the manuscript.
